# *Arabidopsis fad4* mutant analysis provides insights into thermo sensing within plant plasma membrane

**DOI:** 10.3389/fpls.2025.1688284

**Published:** 2026-01-19

**Authors:** Mingjie Chen, Zhenghua Du, Dongsheng Fang, Ming Zou, Jay J. Thelen

**Affiliations:** 1 College of Life Sciences, Xinyang Normal University, Xinyang, Henan, China; 2 Division of Biochemistry and Interdisciplinary Plant Group, Christopher S. Bond Life Science Center, University of Missouri, Columbia, MO, United States; 3 College of Tea and Food Science, Dabie Mountain Laboratory, Xinyang Normal University, Xinyang, Henan, China; 4 Fujian Provincial Key Laboratory of Haixia Applied Plant Systems Biology, Haixia Institute of Science and Technology and School of Life Sciences, Fujian Agriculture and Forestry University, Fuzhou, China

**Keywords:** fatty acid desaturase 4, phosphatidylglycerol, plasma membrane, annexins, thermo sensing, cytosolic free calcium, heat shock, oxidative stress

## Abstract

Increasing evidence suggests that the plasma membrane is the initial site for temperature sensing. In searching for the contributions of membrane lipids to stress-induced cytosolic calcium, we found that *FATTY ACID DESATURASE 4* (*FAD4*) knockout mutant exhibited an elevated acute cytosolic calcium spike in response to heat shock or oxidative stress, but not cold shock. *fad4* mutant plants are more tolerant to heat stress but less tolerant to cold or high light stress. Lipidomic profiling demonstrated that overall phosphatidylglycerol (PG) levels, specifically, PG (36:7) levels, were reduced in the plasma membrane of *fad4* mutants. Based on liposome binding assays, calcium channel proteins annexin2 and annexin4 showed higher association with *fad4* plasma membranes compared to wild type (WT) control. Fat Western analyses indicated that anionic lipids, including phosphatidylserine (PS), phosphatidic acid (PA), and PG, bind to annexin1, 2, 3, and 4 with variable affinity, with PS binding the most tightly and PG the least. These results support the hypothesis that the plasma membrane is the initial site of thermo sensing in *Arabidopsis*.

## 1 Introduction

With global climate change, the frequency and severity of extreme temperatures increase, representing an adaptability challenge for sessile organisms like plants. The deleterious effects of temperature stress on plants are diverse and include membrane leakage (Sangwan et al., 2002; Shomo et al., 2024), protein denaturation (Sharma et al., 2010), and the induction of reactive oxygen species (ROS) (Gong et al., 1998; Volkov et al., 2006). Accordingly, plants undergo a myriad of adjustments in growth and metabolism to defend temperature stress, including reduced growth, membrane lipid remodeling (Alfonso et al., 2001; Sangwan et al., 2002; Saidi et al., 2010; Burgos et al., 2011; Shomo et al., 2024), the induction of heat shock factors (HSFs) and heat shock proteins (HSPs) (Sorger, 1991; Morimoto and Santoro, 1998; Feder and Hofmann, 1999; Busch et al., 2005; Swindell et al., 2007; Schramm et al., 2008; Liu et al., 2011; Liu and Charng, 2013; Li et al., 2019), and the synthesis of detoxifying enzymes and antioxidants to scavenge ROS (Charng et al., 2007; Larkindale and Vierling, 2008; Jung et al., 2013; Chin et al., 2019; Qi et al., 2019).

Temperature sensing in plants may occur at multiple entry points within a signaling pathway, and any cellular component or molecule could potentially act as a thermo sensor (Kerbler and Wigge, 2023). The plasma membrane has long been proposed as a master temperature sensor (Berry and Bjorkman, 1980; Vigh et al., 1993; Ding and Yang, 2022), although the underlying mechanisms are not fully understood. In a previous work, we reported lipid desaturase ADS1 for plant freezing tolerance (Chen and Thelen, 2016). ADS1 desaturates chloroplastic monogalactosyl diacylglycerol (MGDG); its knockout mutants showed lower 34C-species of MGDG than that of wild type (WT). Non-cold acclimated *ads1* mutants exhibited an elevated transient [Ca^2+^]_Cyt_ upon cold shock, and pre-cold acclimation treatment abolished this difference, but *ads1* mutant plants showed enhanced freezing tolerance after cold acclimation (Chen and Thelen, 2016). These data demonstrated that ADS1-mediated chloroplast membrane lipid remodeling primes the cold acclimation response. Biochemical and genetic evidence demonstrate that lipid saturation levels are positively correlated with thermal tolerance of plants (Thomas et al., 1986; Hugly et al., 1989; Kunst et al., 1989; Murakami et al., 2000; Alfonso et al., 2001; Falcone et al., 2004; Chen et al., 2006; Narayanan et al., 2016), and the physical state of membranes controls the signaling mechanism of heat shock (HS) response (Horvath et al., 1998; Sangwan et al., 2002; Saidi et al., 2010). It is generally regarded that membrane lipid compositions could affect the folding, mobility, and activity of integral or membrane-associated proteins (Kunst et al., 1989; Hayes et al., 2021); as a result, these membrane proteins have the potential to sense the changes in membrane state. The plant plasma membrane lipid bilayer is mainly composed of phospholipids, including phosphatidic acid (PA), phosphatidylethanolamine (PE), phosphatidylcholine (PC), phosphatidylserine (PS), phosphatidylinositol (PI), and phosphatidylglycerol (PG) (Lodish et al., 2000). In plant cells, PG can be synthesized from PA in the plastid, endoplasmic reticulum (ER), and mitochondria (Moore, 1982; Xu et al., 2002). The PG synthesized through the plastid pathway (also known as the prokaryotic pathway) contains a *trans*-double bond specific to PG at the *sn*-2 position (16:1^Δ3-trans^-PG or 16:1^t^-PG), which is catalyzed by the FAD4 lipid desaturase (Gao et al., 2009). 16:1^t^-PG is plant-specific, associated with thylakoid membranes, and plays fundamental roles in the photosynthesis and chilling tolerance of plants (Murata, 1983; Murata and Yamaya, 1984; McCourt et al., 1985; Boudière et al., 2014). However, the *fad4* mutation does not significantly affect the stability of chlorophyll–protein complexes to temperature-induced dissociation (McCourt et al., 1985). Recently, Hoh et al. (2022) reported that chilling sensitivity is correlated with the abundance of 16:1^t^-PG in cold-sensitive cowpea as well as cold-hardy *Arabidopsis*. In addition to the prokaryotic pathway for PG synthesis, Hurlock et al. (2014) demonstrated that ER-derived precursors are imported and contribute to plastid PG synthesis. Consistent with this report, Fritz et al. (2007) and Burgos et al. (2011) found a new type of eukaryotic PG in plants, which contains a Δ^3-trans^ in *sn*-2 bound *cis*-unsaturated 18-C fatty acids [abbreviated as PG (18:4t/18:3) or PG (36:7)]. However, its functions remain unclear. Recently, we found that the *fad4* mutation affects both PG (36:7) and 16:1^t^-PG synthesis, and that plant growth and metabolism are also affected, suggesting that FAD4 is also responsible for PG (36:7) synthesis (Chen et al., 2023).

Cytosolic Ca^2+^ is a versatile second messenger; it is generated via Ca^2+^-permeable channels in cellular membranes and then activates downstream genes in response to various stimuli (McAinsh and Pittman, 2009). Ca^2+^-permeable channels are widely distributed in the plasma membrane, vacuole, ER lumen, mitochondrial matrix, chloroplast stroma, and nucleus (Wdowiak et al., 2024). Substantial experimental evidence has demonstrated that cytosolic Ca^2+^ signals are core transducers and regulators in heat shock response (Kudla et al., 2010; Gong et al., 1998; Sangwan et al., 2002). A transient activated Ca^2+^-permeable channel in the plasma membrane is responsible for the transient Ca^2+^ influx (Saidi et al., 2009). Wang et al. (2015) demonstrated that annexins (AtANN1 and AtANN2) mediate calcium influx in response to HS. Knockouts of *atann1* or *atann2* reduce HS-induced [Ca^2+^]_Cyt_, and mutant plants are more sensitive to HS treatment, whereas the overexpression of *AtANN*s enhances heat stress tolerance (Chu et al., 2012). In addition to annexins, cyclic nucleotide-gated channels (CNGCs), including AtCNGC2, AtCNGC4, AtCNGC6, and AtCNGC16, have also been demonstrated to be involved in thermal stress response and innate immunity (Tian et al., 2019; Chin et al., 2013; Finka et al., 2012; Gao et al., 2012; Niu et al., 2020; Tunc-Ozdemir et al., 2013). CNGCs are non-selective monovalent and divalent cation channels (Köhler et al., 1999) and contain a C-terminal cyclic nucleotide (cNMP) binding domain (CNBD) with an overlapping calmodulin-binding domain (CaMBD) and isoleucine–glutamine (IQ) motif (Nawaz et al., 2019). CNGCs are active only when the cyclic nucleotides adenosine 3′,5′-cyclic monophosphate (cAMP) and guanosine 3′,5′-cyclic monophosphate (cGMP) bind to their CNBD (Leng et al., 1999; Balagué et al., 2003; Kaplan et al., 2007; Kudla et al., 2010; Qi et al., 2010; Gao et al., 2012; Wang et al., 2013). Even though genetic and biochemical evidence suggest that certain members of AtANNs mediate Ca^2+^ influx in the heat shock response, it remains unclear how their channel activities are activated by heat stimuli. In this study, we used transgenic aequorin protein as a cytosolic calcium reporter (Alonso et al., 2017). This technique allows real-time monitoring of the dynamic changes of [Ca^2+^]_Cyt_. We found that compared to WT, the *fad4* mutant showed an elevated acute [Ca^2+^]_Cyt_ spike in response to heat shock but not cold shock. Lipidomic profiling revealed that the overall PG levels and specifically PG (36:7) in *fad4* plasma membranes were significantly reduced, calcium channel proteins (AtANN2 and AtANN4) exhibited higher levels of association toward *fad4* plasma membranes, and *fad4* mutants showed enhanced thermal tolerance. These data provide new genetic and biochemical evidence that the plasma membrane is the initial site for early thermal sensing in *Arabidopsis*.

## 2 Materials and methods

### 2.1 Plant materials

Two independent T-DNA insertion lines in the *FAD4* gene were obtained from the *Arabidopsis* Biological Resource Center (Ohio, USA), including *fad4-2* (stock name CS878899) and *fad4-3* (stock name CS874467). Biochemical analysis demonstrated that both T-DNA insertion lines showed a similar change in the gas chromatography profile of 16:1^t^ as the *fad4–1* mutant. The *fad4–1* is an Ethyl methyl sulfonate (EMS) mutant with a point mutation converting 177 Trp into a stop codon, thus deactivating the FAD4 protein (Gao et al., 2009). Also, RT-PCR was applied, and both *fad4–2* and *fad4–3* were verified as knockout mutants. Plants were grown in composite soil with peat soil:vermiculite:perlite (3:1:1, v/v/v). The growth chamber conditions were as follows: photoperiod, 16 h light/8 h dark; temperature, 22 °C; light intensity, 160 μmol photon m^−2^ s^−1^; and humidity, 60%.

### 2.2 Temperature stress treatment

*Col*, *fad4-2*, and *fad4–3* were first germinated in Murashige–Skoog (MS) medium for 1 week; then, the seedlings were transferred into soil pots and grown at three individual growth chambers with variations only in temperature setting at 10 °C, 22 °C, and 28 °C. The photoperiod was 12 h light/12 h dark; the normal light and high light intensity were ~160 and ~670 μmol photon m^−2^ s^−1^, respectively. Plants were photographed after growing for two additional weeks.

### 2.3 Fatty acid analysis

*Col* and *fad4–2* were grown in two growth chambers with temperature settings at 22 °C and 28 °C, respectively. After growing for 3 weeks, plants were harvested and derivatized with acid methanol. The Fatty acid methyl ester (FAMEs) were extracted twice with hexane, combined and concentrated, and then analyzed via Gas Chromatography-Mass Spectrometry (GC-MS) and Gas Chromatography-Flame Ionization Detector (GC-FID) (GC-MS QP2010 plus, Shimadzu, Suzhou, China). One microliter of the sample was injected in a 10:1 split ratio and separated using the RT-2560 column (0.25 mm × 30 m × 0.25 μm). Helium was used as carrier gas with a flow rate of 1 mL min^−1^. Oven temperature was programmed as follows: initiated at 150 °C, ramped to 200 °C at 2 °C min^−1^, and held for 5 min. The flow rates of nitrogen, hydrogen, and air were set as 30, 40, and 400 mL min^−1^, respectively. The temperatures for the injector, Flame Ionization Detector (FID), ion source, and interface were 240 °C, 220 °C, 200 °C, and 230 °C, respectively. Fatty acids were identified using Quest mass spectra with the National Institute of Standards and Technology database (NIST14), and their contents were calculated from the FID dataset by normalizing respective peak area to the internal standard.

### 2.4 *fad4* mutant complementation and overexpression of *FAD4* gene

*FAD4* CDS was amplified from WT total RNA using RT-PCR. The gene-specific primer pairs were integrated with *Bam*HI and *Xho*I recognition sequences: 5′-CGGGATCCATGGCTGTATCACTTCC-3′ and 5′-CCGCTCGAGTTATGCTTGGTTGTTGG-3′. The amplified fragment was digested with *Bam*HI and *Xho*I and then cloned into the pMGmubi binary vector, in which the *FAD4* transgene was driven by a soybean ubiquitin promoter. The construct was introduced into the *Agrobacterium tumefaciens* strain GV3101 and then transformed into the *fad4–2* mutant for complementation analysis. The same construct was transformed into wild-type *Col* to generate *FAD4* overexpression lines. Transgenic plants were identified and propagated; homozygous transgenic T3 plants were used for experiments. RT-PCR was applied to confirm transcriptional complementation of *fad4* mutant and *FAD4* overexpression (**Supplementary Figure S1**).

### 2.5 Real-time PCR

Total plant RNA was isolated using an RNAprep pure plant plus kit (Tiangen, Beijing, China), and the cDNA was synthesized using M-MLV reverse transcriptase (Promega, Madison, WI, USA). To analyze gene expression, gene-specific primer pairs were designed (**Supplementary Table S1**). Quantitative real-time PCR (RT-qPCR) was performed on the CFX96 system (Bio-Rad, Hercules, CA, USA) using the TB Green^®^ Premix Ex Taq II kit (Takara, Dalian, Japan), and three biological replicates were applied. The RT-qPCR conditions were 95 °C for 30 s, followed by 40 cycles at 95 °C for 5 s and 60 °C for 30 s. The melting curve conditions were 95 °C for 15 s, 60 °C for 1 min, and 95 °C for 15 s. The relative transcript abundance was calculated using the 2^−ΔΔCT^ method. *AtUBQ* was used as the internal reference gene.

### 2.6 Double mutant construction

A cytosolic apoaequorin transgene in the *Columbia* background [aequorin (AEQ) line] was used as a [Ca^2+^]_Cyt_ reporter. To integrate the *fad4* mutant locus into the AEQ line, *fad4–2* was crossed with AEQ. F1 plants were self-pollinated to obtain F2 seeds, and individual F2 plants were screened via PCR genotyping to identify homozygous double mutants AEQ:*fad4-2*.

### 2.7 [Ca^2+^]_Cyt_ measurement

[Ca^2+^]_Cyt_ measurement followed previously established laboratory protocol (Chen and Thelen, 2016). Briefly, AEQ and AEQ:*fad4–2* seeds were germinated vertically on half-strength Murashige–Skoog media at 22 °C under continuous light (20 μmol m^−2^ s^−1^). One-week-old seedlings were transferred to a 96-well microplate, and eight biological replicates were used. Seedlings were incubated in 50 μL of reconstitution buffer (2 mM 2-Morpholinoethanesulphonic acid (MES), 10 mM CaCl_2_, and 2.5 μM coelenterazine h) overnight. The next day, the microplate was loaded into the Verita microplate luminometer (Promega, Madison, WI, USA) to measure photon emission in kinetic mode. A background reading was first recorded for 10 s, and then stress stimuli were applied. For heat shock and cold shock treatments, water at 42 °C and 0 °C, respectively, was applied; for oxidative stress treatment, 10 mM H_2_O_2_ was used. After 100 μL of such a solution was injected into one well, photon emission was immediately recorded for 60 s at a rate of one read per second, then 150 μL of discharge buffer (2 M CaCl_2_, 20% ethanol) was injected into the same well, and photon emission was recorded for an additional 60 s. The calibration of luminescence values to calcium concentrations followed the method described by Knight et al. (1996).

For inhibitor treatment, GdCl_3_ and ruthenium red were used as a Ca^2+^ influx inhibitor and an intracellular Ca^2+^ channel blocker, respectively (Knight et al., 1992). After being transferred into a 96-well microplate, seedlings were divided into three groups: control, GdCl_3_ treatment, and ruthenium red treatment. A 250-μL volume of 75 μM GdCl_3_ or 25 μM ruthenium red (Sigma-Aldrich, St. Louis, MO, USA) was added to immerse the seedling completely, 250 μL of water was added to control plants, and each group had eight biological replicates. The microplate was incubated in the dark for 3 h, and the solution was removed as much as possible before adding the reconstitution buffer.

### 2.8 Recombinant annexin protein expression and purification

The coding sequences of *AtANN1*, *AtANN2*, *AtANN3*, and *AtANN4* were amplified from *Col* total RNA using RT-PCR. *Eco*RI and *Xho*I cutting sites were integrated into gene-specific forward and reverse primer pairs, except for the *AtANN4* reverse primer, which was integrated with the *Sal*I cutting site (**Supplementary Table S2**). The PCR products were cloned into the pEASY-BLUNT vector (Transgen Biotech, Beijing, China); the inserts were dropped off by corresponding double enzyme digestion and then cloned into the pET28a expression vector. The reconstructed plasmids were introduced into *Escherichia coli* Transetta (DE3) competent cells (Transgen Biotech, Beijing, China) for protein expression. Recombinant proteins were purified using BeyoGold His-tag Purification Resin (Beyotime, Nantong, Jiangsu, China) under denaturing conditions (**Supplementary Figure S2**). To renature proteins, denatured proteins were sequentially dialyzed in Tris Base/Sodium Chloride (TBS) buffer containing 6, 4, 2, and 0 M urea, each step lasting for 12 h. Protein concentration was determined using Pierce™ 660 nm protein assay reagent (Thermo Scientific, Rockford, IL, USA).

### 2.9 Fat Western blotting

Fat Western blotting followed the method described by Stevenson et al. (1998). 1,2-Dioleoyl-*sn*-glycero-3-phospho-rac-(1-glycerol) (PG), 1-palmitoyl-2-oleoyl-*sn*-glycero-3-phosphocholine (PC), 1-palmitoyl-2-oleoyl-*sn*-glycero-3-phosphoethanolamine (PE), and 1,2-diacyl-*sn*-glycero-3-phospho-l-serine (PS) were purchased from Sigma (St. Louis, MO, USA); 1,2-dioleoyl-*sn*-glycero-3-phospho-(1′-myo-inositol) (PI), 1-palmitoyl-2-oleoyl-*sn*-glycero-3-phosphate (PA), MGDG, and digalactosyl diacylglycerol (DGDG) were obtained from Avanti (Shanghai, China). Lipid standards were dissolved in chloroform at 1 mg mL^−1^; 0.5, 1.0, or 5.0 μg was spotted onto nitrocellulose membrane (Merck Millipore, County Cork, Ireland) and dried at room temperature for 1 h; then, the membrane was incubated in Tris Base/Sodium Chloride/Tween 20 (TBST) buffer [10 mM Tris (pH 8.0), 140 mM NaCl, and 0.1% (v/v) Tween 20] containing 3% (w/v) delipidated bovine serum albumin for 1 h, followed by overnight incubation in 10 mL TBST solution containing renatured His-tagged AtANNs (0.5 μg mL^−1^) at 4 °C. The membrane washing, incubation with primary antibody, and membrane staining followed the routine Western blotting protocol.

### 2.10 Plasma membrane purification

Plasma membrane purification followed the method described previously (Larsson et al., 1987; Chen et al., 2020). *Col*, *fad4-2*, and *fad4* complementation line (*CO-fad4*) were grown in chamber for 3 weeks; ~40-g leaves were harvested and homogenized in 160 mL of buffer for 1 min with a kitchen homogenizer (JYL-C91T, Jiuyang, Jinan, Shandong, China); the buffer contained 330 mM sucrose, 50 mM Tris-MES (pH 7.8), 5 mM Ethylenediaminetetraacetic acid (EDTA), 5 mM Dithiothreitol (DTT), 0.5 mM Phenylmethylsulfonyl fluoride (PMSF), 0.2% (w/v) bovine serum albumin (BSA), 0.6% (w/v) Polyvinylpyrrolidone (PVP), and 5 mM ascorbic acid. The homogenate was filtered through 240-μm nylon cloth and then centrifuged at 10,000 *g* for 10 min (Beckman Coulter Allegra X-30R). The supernatant was centrifuged at 50,000 *g* for 30 min to obtain a microsomal pellet (Beckman Coulter Optima XPN-100, Brea, CA, USA); then, the pellet was suspended in buffer containing 330 mM sucrose, 5 mM potassium phosphate (pH 7.8), 5 mM KCl, 1 mM DTT, and 0.1 mM EDTA. Nine grams of the suspension was loaded into a pre-prepared 27.0-g phase mixture, thoroughly mixed, then centrifuged at 1,500 *g* for phase separation. The phase separation was repeated three times. The upper phase was combined and diluted >2-fold with suspension buffer and then centrifuged at 100,000 *g* for 60 min. The supernatant was discarded; the pellet contained purified plasma membrane. To isolate lipids from purified plasma membrane, the Bligh and Dyer (1959) method was applied.

### 2.11 Lipidomic profiling

Lipid samples were analyzed on an electrospray ionization tandem mass spectrometer (ESI-MS/MS) (Waters Xevo TQS mass spectrometer; Waters Corporation, Milford, MA, USA), and sequential precursor and neutral loss scans were applied as described previously (Shiva et al., 2013). Phospholipid internal standards were used for individual lipid class identification and quantification (Welti et al., 2002). Internal standards and their acquisition information are provided in Chen et al. (2023); PG (36:7) identification was detailed in Fritz et al. (2007).

### 2.12 Liposome preparation and protein association assay

Liposome preparation followed the method described by Wang et al. (2012), with minor modifications. Purified plasma membrane lipid (250 μg) was suspended in 0.5 mL TBS buffer containing 0.2 M choline chloride, hydrated at 37 °C for 1 h, and then vortexed vigorously for 2 min; the resulting multi-lamellar vesicles were centrifuged at 13,000 × *g* for 10 min and washed once with 0.5 mL TBS buffer containing 0.2 M choline chloride. The liposome was suspended into 100 μL TBS buffer containing 0.2 M choline chloride; purified renatured recombinant annexin protein (2.0 μg) was added, incubated on ice for 30 min, and then centrifuged at 13,000 *g* for 10 min; the pellet was washed twice with 500 μL TBS containing 0.2 M choline chloride; the protein–liposome pellet was suspended in 20 μL 2× Sodium dodecyl sulfate polyacrylamide gel electrophore (SDS–PAGE) loading dye, loaded, and separated on SDS–PAGE; separated proteins were transferred onto Polyvinylidene Fluoride (PVDF) membrane for Western blotting.

For Western blotting, the PVDF membrane was incubated in 1× TBST containing 5% milk at room temperature for 1 h and washed five times with 1× TBST and 5 min per wash. Mouse anti-6x His tag monoclonal antibody (Horseradish peroxidase (HRP)-conjugated; Abmart, Shanghai, China) was diluted 5,000-fold, and the membrane was incubated at 4 °C overnight. The membrane was washed three times for 10 min per wash and then incubated with chemiluminescent HRP substrate (Millipore, Burlington, MA, USA). The signals were captured using a luminescent imaging system (Tanon 5200, Tanon Technology Co., Ltd., Shanghai, China).

### 2.13 Statistical analysis

The average and standard errors were calculated using the Excel software. Significance was determined using one-way ANOVA based on Duncan’s multiple range tests in the SPSS software.

## 3 Results

### 3.1 *fad4* mutants showed elevated cytosolic calcium spike in response to heat shock but not cold shock

**Figure 1:**
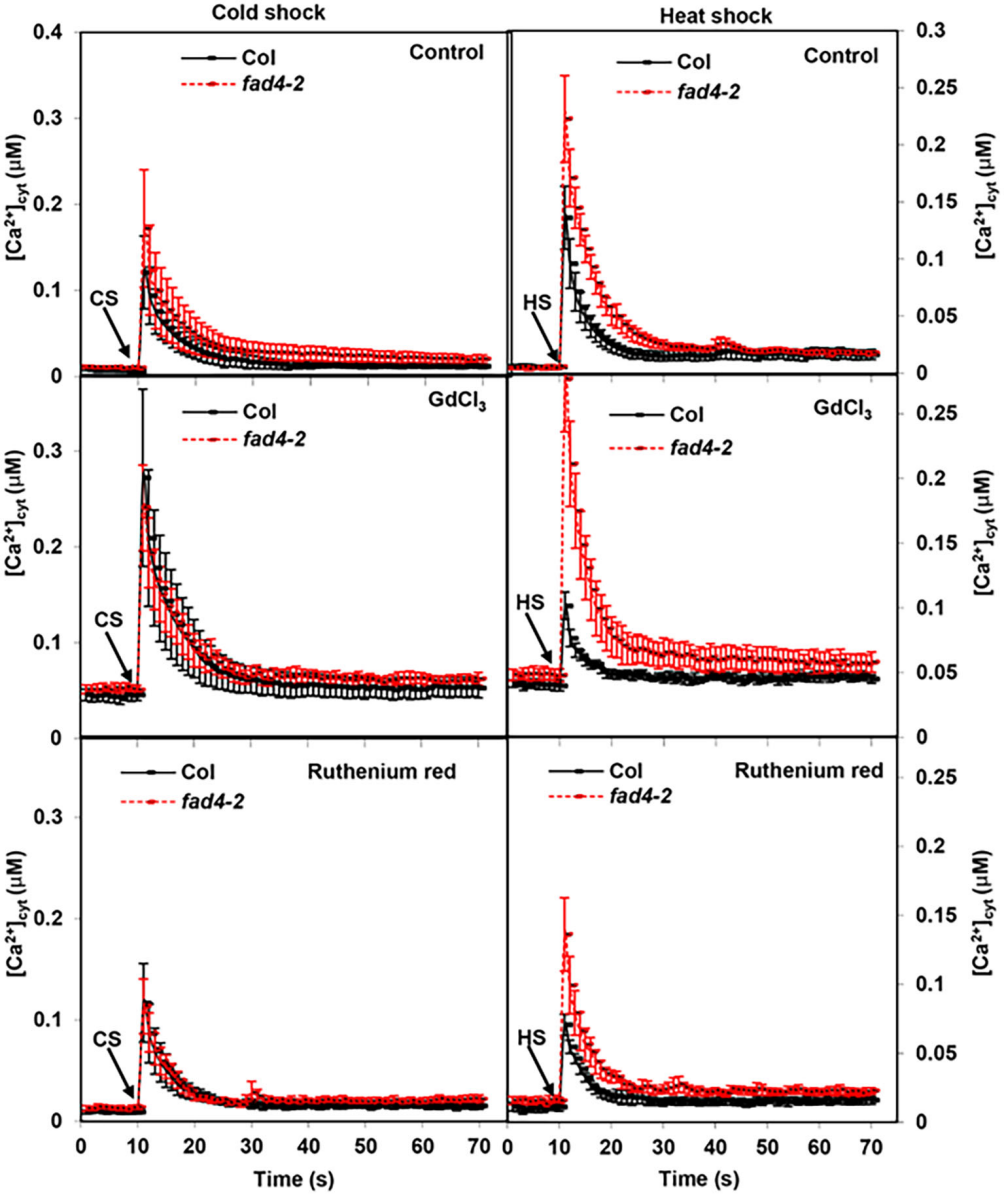
Effect of cold and heat shock on cytosolic calcium levels in wild type (WT) and *fad4–2* mutant. Cold shock (CS) or heat shock (HS) was applied at 10 s as indicated by the arrow. Aequorin (AEQ) and AEQ:*fad4–2* seeds were germinated vertically on 0.5× Murashige–Skoog (MS) medium at 23 °C under continuous light, and 7-day-old seedlings were used for calcium measurement. Plants were divided into three groups and pretreated with water (control), 75 μM GdCl_3_, and 25 μM ruthenium red. Data were expressed as average ± standard error (n = 8).

Membrane fluidity changes have been suggested to be the earliest event in temperature sensing (Mittler et al., 2012; Bourgine and Guihur, 2021), even though the underlying molecular details await clarification. Since *acyl-lipid desaturases* (*ADS*s) and *fatty acid desaturases* (*FAD*s) catalyze lipid desaturation leading to membrane fluidity alteration (Fukuchi-Mizutani et al., 1995; Shomo et al., 2024; Yu et al., 2021; Higashi and Saito, 2019), we reasoned that *ads* or *fad* mutants could potentially affect early events of temperature sensing, such as [Ca^2+^]_Cyt_. To test this hypothesis, the transgenic AEQ line (Knight et al., 1996) was utilized as [Ca^2+^]_Cyt_ reporter, and various *ads* or *fad* mutant loci were crossed into this reporter line, permitting stress-induced, early, live [Ca^2+^]_Cyt_ comparison between WT and *ads* or *fad* mutants. To differentiate the origin of Ca^2+^, GdCl_2_ and ruthenium red were respectively applied to block calcium influx from the apoplast or intracellular calcium release. Our time-lapse recording focused on early [Ca^2+^]_Cyt_ changes after stimulation in an attempt to find upstream signaling components. The resting [Ca^2+^]_Cyt_ levels were first recorded for 10 s before stimulation, then stimuli were applied, and signals were immediately recorded for 60 s. This screening led us to identify *fad4* with an altered [Ca^2+^]_Cyt_ signature in response to heat shock but not cold shock. The resting [Ca^2+^]_Cyt_ levels in control WT and *fad4–2* seedlings were 0.007 and 0.011 μM, respectively; upon cold shock (CS), [Ca^2+^]_Cyt_ amplitude reached 0.12 and 0.17 μM within 1 s, followed by a rapid decay (**Figure 1**, left upper panel). When seedlings were pretreated with GdCl_3_ to block Ca^2+^ entry from apoplast, upon cold shock, [Ca^2+^]_Cyt_ amplitude elevated to 0.27 and 0.24 μM within 1 s in WT and *fad4-2*, respectively (**Figure 1**, left middle panel). When seedlings were pretreated with ruthenium red, upon cold shock, [Ca^2+^]_Cyt_ levels reached 0.12 and 0.11 μM within 1 s in WT and *fad4-2*, respectively (**Figure 1**, left lower panel). These data indicate that CS can induce an acute [Ca^2+^]_Cyt_ spike, which was not significantly affected by *fad4* mutation. Pretreatment with Gd^3+^ enhanced both resting and CS-induced acute [Ca^2+^]_Cyt_ spike (**Figure 1**, left middle panel), suggesting that blocking apoplastic calcium influx by Gd^3+^ pretreatment could potentially enhance intracellular calcium release, or Gd^3+^ is not effective at blocking CS-induced [Ca^2+^]_Cyt_ elevation.

For HS treatment, in control WT and *fad4–2* plants, HS induced an acute [Ca^2+^]_Cyt_ spike of 0.14 μM and 0.22 μM within 1 s (**Figure 1**, right upper panel), indicating that the *fad4* mutation elevated the [Ca^2+^]_Cyt_ spike in response to HS. Unlike cold shock, Gd^3+^ pretreatment showed diverse effects on HS-induced acute [Ca^2+^]_Cyt_ elevation in WT and *fad4–2* mutant plants: WT showed an acute [Ca^2+^]_Cyt_ amplitude of 0.10 μM, which was 29% lower compared to that of control WT, while this acute [Ca^2+^]_Cyt_ amplitude in *fad4–2* was increased to 0.28 μM, which was 27% higher compared with that of control *fad4-2* (**Figure 1**, right middle panel), indicating that the *fad4* mutation abolished the blockage effect of Gd^3+^ on HS-induced calcium entry from apoplast. When seedlings were pretreated with ruthenium red, the acute [Ca^2+^]_Cyt_ peak in WT was reduced to 0.07 μM, which corresponded to a 50% decrease compared to that of control, while this [Ca^2+^]_Cyt_ peak in *fad4–2* was reduced to 0.14 μM, which corresponded to a 36% decrease, indicating that the HS-induced intracellular Ca^2+^ release in *fad4* was normal as the WT control.

### 3.2 *fad4* mutation resulted in elevated [Ca^2+^]_Cyt_ spike in response to H_2_O_2_

**Figure 2:**
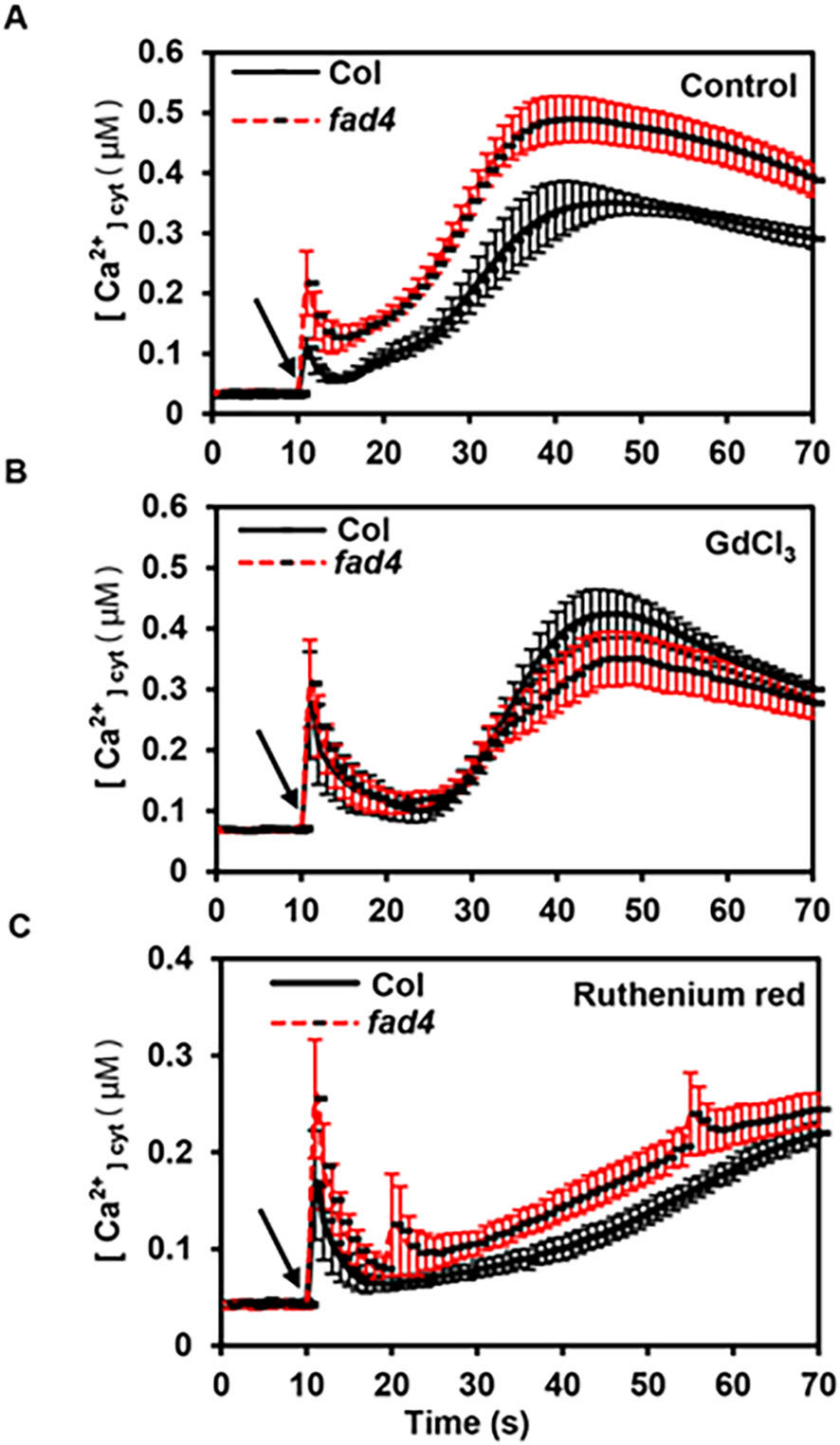
Effect of H_2_O_2_ on cytosolic calcium levels in wild type (WT) and *fad4–2* mutant. H_2_O_2_ was applied at 10 s as indicated by arrow. Aequorin (AEQ) and AEQ:*fad4–2* seeds were germinated vertically on 0.5× Murashige–Skoog (MS) medium at 23 °C under continuous light, and 7-day-old seedlings were used for calcium measurement. Plants were divided into three groups and pretreated with water (control) **(A)**, 75 μM GdCl_3_ **(B)**, and 25 μM ruthenium red **(C)**.

Peroxiredoxin Q (Prx Q) converts H_2_O_2_ to H_2_O (Lamkemeyer et al, 2006); it is an interaction partner with FAD4 *in planta* (Horn et al., 2020). The *Arabidopsis prxq* T-DNA mutants showed altered sensitivity to oxidants (Lamkemeyer et al, 2006), which prompted us to investigate whether the *fad4* mutation affects its response to oxidative stress. We found that 10 mM H_2_O_2_ treatment induced biphasic [Ca^2+^]_Cyt_ increase in both WT and *fad4* mutant plants: the first small peak appeared within 1 s after H_2_O_2_ application, followed by rapid decay for 5 s; then, the peak increased again to form the second prolonged large peak at 36 s following H_2_O_2_ treatment; a slow signal decay then ensued (**Figure 2A**). Although this biphasic pattern was not altered by *fad4* mutation, the amplitudes from the *fad4–2* mutant were higher than those of WT, especially for the first peak [0.22 μM (*fad4*) vs. 0.11 μM (WT)] (**Figure 2A**). When the calcium influx was blocked by GdCl_3_ pretreatment, this biphasic peak pattern was retained in WT and *fad4-2*. However, [Ca^2+^]_Cyt_ amplitude differences of both peaks between WT and the *fad4* mutant were largely abolished [the first peak, 0.27 μM (WT) vs. 0.3 μM (*fad4*); the second peak, 0.42 μM (WT) vs. 0.35 μM (*fad4*)] (**Figure 2B**). Unexpectedly, compared to the untreated control, Gd^3+^ pretreatment even increased the amplitudes of the first peak, especially in WT plants (0.27 vs. 0.11 μM) compared to the *fad4–2* mutant (0.3 vs. 0.22 μM) (**Figure 2B**). This suggests that the blockage of Ca^2+^ entry from apoplast could enhance intracellular Ca^2+^ release in response to H_2_O_2_ stimuli. Ruthenium red pretreatment slightly elevated the first small peak [0.17 μM (WT) vs. 0.26 μM (*fad4*)], the second large peak showed a slower increase, and their difference became smaller (**Figure 2C**). These data suggest that H_2_O_2_ treatment first induced a transient calcium entry from apoplast, which formed the first small peak; this transient [Ca^2+^]_Cyt_ increase may activate intracellular calcium release, leading to the formation of the second large peak. This could explain why Gd^3+^ pretreatment not only abolished the first peak difference but also abolished the second peak difference (**Figure 2B**). Price et al. (1994) also reported that H_2_O_2_ treatment induced [Ca^2+^]_Cyt_ biphasic changes in tobacco.

### 3.3 *fad4* mutation enhanced heat tolerance but reduced cold tolerance

**Figure 3:**
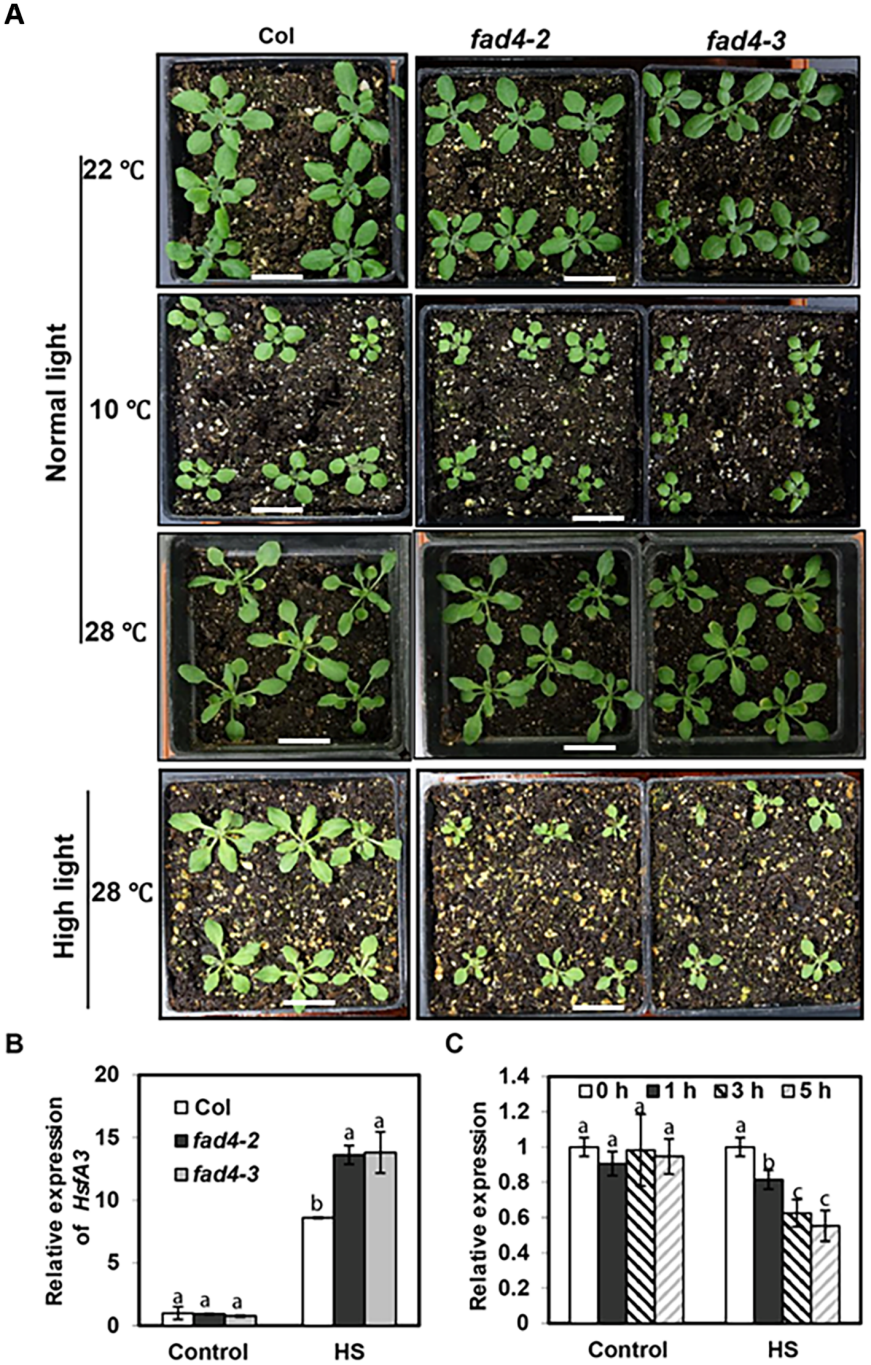
*fad4* mutant phenotype grown under different temperatures and light intensities. **(A)** Wild type (WT), *fad4-2*, and *fad4–3* were grown at 22 °C, 10 °C, and 28 °C, respectively. Seeds were germinated on Murashige–Skoog (MS) plate; 1-week-old seedlings were transplanted into soil pots and grown in 22 °C growth chamber under 12 h light/12 h dark for 7 days; then, part of the plants were transferred into 10 °C and 28 °C growth chambers and allowed to grow for additional 2 weeks before being photographed. Bar = 2 cm. **(B)** *HsfA3* was upregulated by *fad4* mutation in response to heat shock treatment. Seeds were germinated on 0.5× MS medium for 7 days at 22 °C under continuous light, then were moved to 37 °C growth chamber for 1 h, and recovered at 22 °C growth chamber for 2 h before tissue harvesting. RT-qPCR was applied to monitor gene expression, and data were expressed as mean ± SE (n = 3). **(C)** *FAD4* transcription was inhibited by heat shock treatment. *Col* seedlings were germinated in 0.5× MS liquid medium under hydroponic conditions for 2 weeks, and the seedlings were transferred into fresh 0.5× MS liquid medium. For heat stress treatment, the seedlings were moved into 37 °C growth chamber, while the control plants were held in 22 °C growth chamber; seedlings were harvested at 0, 1, 3, and 5 h after the initiation of heat stress treatment. RT-qPCR was applied to measure *FAD4* transcription levels, and data were expressed as mean ± SE (n = 3). HS, heat stress. Different lowercase letters in the same treatment group represent statistically significant differences.

To test whether HS-induced early acute [Ca^2+^]_Cyt_ elevation in the *fad4* mutant was correlated with growth performance under temperature stress, wild type (*Col*) and two independent *fad4* mutant lines (*fad4-2* and *fad4-3*) were each grown at 10 °C, 22 °C, and 28 °C in combination with normal light and high light. Under normal light intensity (~160 μmol photon m^−2^ s^−1^), whether plants were grown at normal temperature (22 °C) or chilling temperature (10 °C), both *fad4* mutant lines were visibly smaller than the WT control (**Figure 3A**, upper and middle panels), indicating that *fad4* mutation enhances cold-induced growth inhibition compared to WT. Under mild higher temperature (28 °C), these growth differences largely disappeared (**Figure 3A**, middle panel), indicating that the *fad4* mutation enhanced its tolerance to mild higher temperature stress. However, when a 28 °C growth temperature was coupled with higher light intensity (~670 μmol photon m^−2^ s^−1^), both *fad4* mutant lines grew much smaller compared to the WT control (**Figure 3A**, lower panel), suggesting that the *fad4* mutation enhanced their sensitivity to high light stress.

To further investigate the heat tolerance phenotype of *fad4* mutant plants, the expression levels of heat-responsive transcriptional factors (*HSF*s), including *HsfA1a*, *HsfA1b*, *HsfA1d*, *CBK3*, *HsfA2*, *HsfA3*, and *ROF1*, were compared between WT and *fad4* mutants before and after HS treatment. Under 22 °C growth temperature, the expression levels of *HsfA3* from both *fad4* mutant lines were similar to those of the WT control. However, upon HS treatment for 1 h, *HsfA3* expression levels in *fad4* were significantly higher than those of the WT control (**Figure 3B**). The transcript abundance of other *HSF*s between WT and *fad4* mutants did not show consistently significant differences following HS treatment (**Supplementary Figure S3**). HsfA3 regulates multiple heat-responsive genes; its expression levels are associated with plant tolerance to heat (Yoshida et al., 2008; Jung et al., 2013). Our data suggest that *HsfA3* could specifically function downstream of FAD4-mediated HS response.

To observe whether the expression of the *FAD4* gene itself could be regulated by HS, hydroponically cultured WT seedlings were subjected to 22 °C or 37 °C treatments for variable times. RT-qPCR results demonstrated that in control plants (22 °C), the transcriptional levels of *FAD4* remained constant. However, *FAD4* expression was significantly decreased starting at 1 h following 37 °C treatment (**Figure 3C**), indicating that higher temperature suppresses *FAD4* gene transcription.

### 3.4 *fad4* mutant plants retained capacity of lipid saturation regulation in response to changing temperature

To test whether the growth phenotypes of *fad4* (**Figure 3**) were associated with membrane dysfunction in fluidity regulation in response to temperature shift, WT and *fad4–2* mutant plants were grown at 22 °C or 28 °C, and the fatty acid composition was analyzed and compared. Under either 22 °C or 28 °C growth temperature, the 16:0 content of *fad4–2* mutant plants was significantly higher, and the 16:1 content was lower than that of the WT control (**Table 1**), and this is consistent with FAD4’s biochemical function (Gao et al., 2009). Compared to those at 22 °C growth temperature, WT plants grown at 28 °C showed higher 16:0, 16:1, 18:0, 18:1, and 18:2 but lower 16:3 and 18:3 (**Table 1**), confirming that in response to temperature increase, WT plants increased their membrane lipid saturation levels. In our current GC-MS analysis, 16:1^Δ7^ and 16:1^Δ3t^ coelute, so we count them together as 16:1. Comparing 28 °C to 22 °C growth temperature, 16:1 content was higher in WT and *fad4–2* mutant (**Table 1**). The increase in 16:1 in WT plants grown at 28 °C was likely due to an increase in 16:1^Δ7^ rather than 16:1^Δ3t^ for the following considerations: 1) higher growth temperature downregulated *FAD4* gene expression (**Figure 3C**), which could lead to reduced 16:1^Δ3t^ synthesis, and 2) *fad4–2* knockout mutant also showed higher 16:1 level when grown under 28 °C compared to 22 °C (**Table 1**). Interestingly, the *fad4–2* mutant plants showed similar fatty acid compositional changes as WT in response to rising temperature (**Table 1**). Thus, *fad4–2* mutant plants retained this capacity as the WT control.

**Table 1:** Leaf FAME compositional changes of *Col* and *fad4–2* grown under different temperatures.

FAMEs	*Col*		*fad4-2*	
	22 °C	28 °C	22 °C	28 °C
16:0	19.95 ± 1.17	23.95 ± 1.73^#^	24.10 ± 2.60^*^	27.44 ± 0.82^*,#^
16:1^Δ7^/16:1^Δ3t^	7.16 ± 0.55	7.98 ± 0.53^#^	1.35 ± 0.16^*^	3.02 ± 0.08^*,#^
16:2	1.09 ± 0.08	1.08 ± 0.08	0.65 ± 0.56	1.07 ± 0.04
16:3	13.9 ± 0.28	10.68 ± 0.71^#^	14.66 ± 0.19^*^	10.90 ± 0.15^#^
18:0	1.54 ± 0.21	2.40 ± 0.18^#^	1.46 ± 0.23	2.50 ± 0.29^#^
18:1	5.00 ± 0.28	5.94 ± 0.71^#^	6.11 ± 0.19^*^	6.99 ± 0.15^#^
18:2^Δ9,12^	21.89 ± 1.03	25.61 ± 0.30^#^	21.72 ± 0.83	26.35 ± 0.88^#^
18:3^Δ9,12,15^	29.50 ± 2.00	22.35 ± 1.50^#^	29.95 ± 3.00	21.75 ± 1.15^#^

Data were expressed as mean ± standard deviation (n = 3). Statistically significant differences (p < 0.05) between wild type (WT) and *fad4–2* mutant under same growth temperature are represented by asterisk (*). Statistically significant differences (p < 0.05) between 22 °C and 28 °C of same plants are represented by #.

### 3.5 Plasma membrane PG levels were reduced in *fad4* mutant

**Figure 4:**
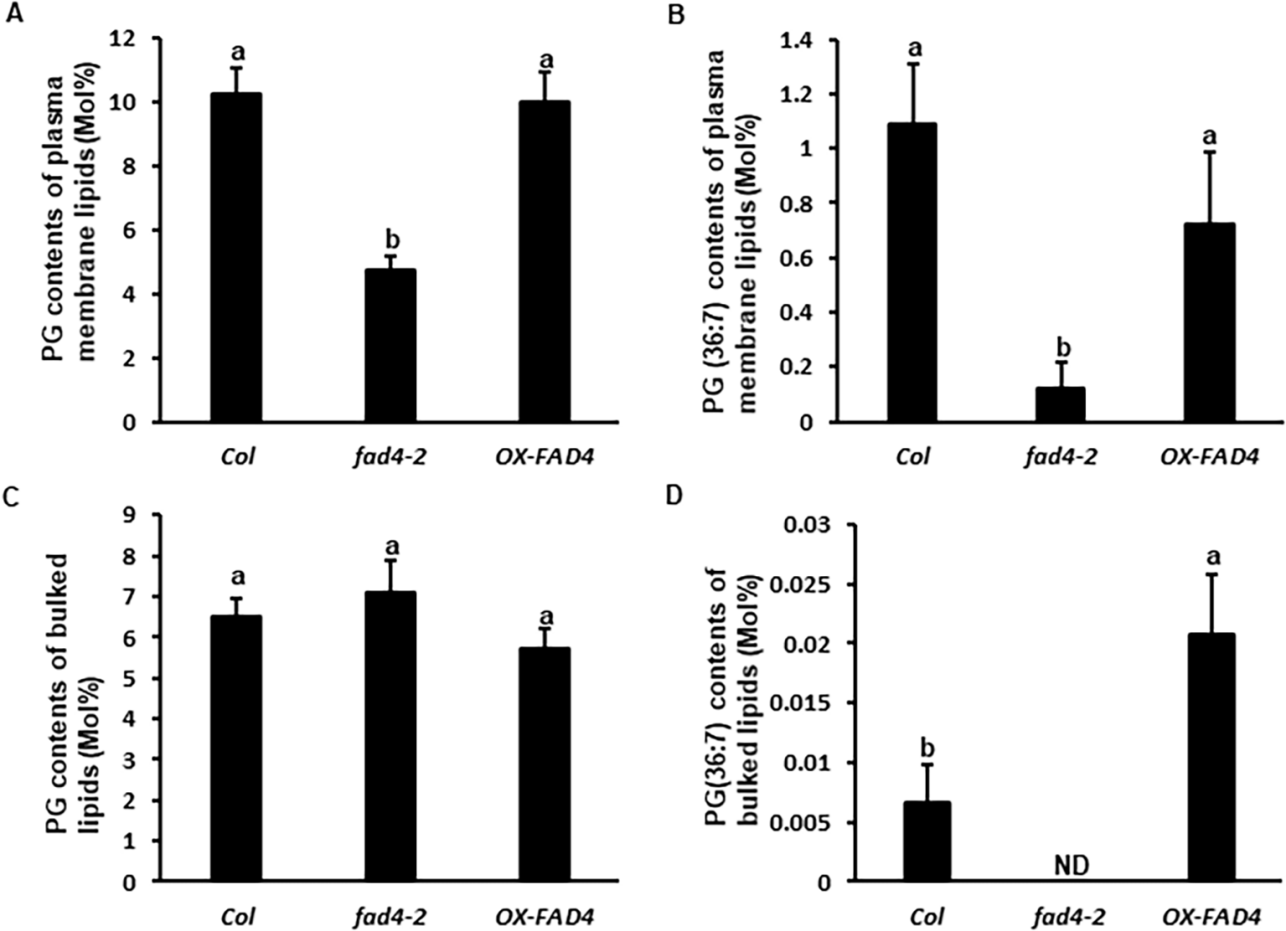
The effect of knockout and overexpression of *FAD4* gene on plasma membrane PG levels. Plasma membrane was purified from *Col*, *fad4-2*, and *OX-FAD4* plants by two-phase separation. Total plasma membrane lipids were isolated from purified plasma membrane; total bulked lipids were isolated from whole plants. Lipids were analyzed by electrospray ionization tandem mass spectrometer using sequential precursor and neutral loss scans. Four biological replicates were applied, and data were expressed as mean ± standard deviation (n = 4); different lowercase letters represent statistically significant differences.

Live [Ca^2+^]_Cyt_ data demonstrated that the *fad4* mutation enhanced calcium channel activity (**Figures 1**, **2**), which could be associated with the altered lipid composition of the plasma membrane. To test this hypothesis, plasma membrane lipids from *Col*, *fad4-2*, and *FAD4* overexpression line (*OX-FAD4_2*) were purified using the two-phase separation method (Larsson et al., 1987; Chen et al., 2020). Meanwhile, whole leaf lipids were also extracted, and both types of total lipid samples were analyzed and compared via lipidomic profiling. In purified plasma membranes, the total PG fraction of *fad4–2* mutant plants was reduced to approximately one-half of WT or *OX-FAD4_2* plants (**Figure 4A**); specifically, PG (36:7) was represented at ~11% of the WT level (**Figure 4B**). It was unexpected that this minor fraction of PG (36:7) was also detected in the *fad4–2* plasma membrane, in view that *fad4–2* is a knockout mutant. We speculate that some uncharacterized genes in the *Arabidopsis* genome may show minor FAD4-like activities, and their products could be channeled into the plasma membrane. The *FAD4* gene has two closely related paralogs in *Arabidopsis*, At1g62190 and At2g22890; however, their functions remain unclear. The overexpression of *FAD4* did not significantly alter plasma membrane total PG and PG (36:7) levels compared to the WT control (**Figures 4A, B**). For whole leaf lipid extracts, total PG levels were not significantly affected in either *fad4* knockout plants or *OX-FAD4* lines (**Figure 4C**). However, the minor PG (36:7) lipid species was undetectable in the *fad4–2* mutant plants, while its content in *OX-FAD4_2* was significantly higher compared to that of the WT control (**Figure 4D**). To estimate the purity of plasma membrane (PM) samples, we focused on two most abundant galactolipids that are known to be found in chloroplast: MGDG (34:6) and DGDG (36:6). In whole plant lipid extract, MGDG (34:6) and DGDG (36:6) account for 34–36 and 11 mol%, respectively; in PM lipids, both account for 0.4–0.7 and 0.6–1.5 mol%, respectively (**Supplementary Material 2**). These data suggest that our PM preparations contained <10% contamination from chloroplast membrane, which is comparable to our previous observation (Chen et al., 2020). The isolation of the plasma membrane had purging effects on the chloroplast membrane. Thus, in PM samples, lipids located on the chloroplast were diluted, while lipids located on PM were enriched. To reflect these lipid compositional changes, here, the enrichment factor, the ratio of a lipid’s mol% in the plasma membrane to its mol% in whole plant lipids, was calculated. If PG (36:7) were exclusively located in the chloroplast, we would expect that its enrichment factor could show similar changes as MGDG and DGDG, which are <1 (**Supplementary Material 2**). However, its enrichment factors in *Col* and *OX-FAD4* were 165 and 35, respectively, in soil-grown plants; this result did not agree with the notion that PG (36:7) is solely located in the chloroplast membrane. We reasoned that a sound explanation for this result is that PG (36:7) is also present in the plasma membrane. Fritz et al. (2007) reported its presence in chloroplasts, which raises the question of how PG (36:7) is transported from its synthetic site to the plasma membrane. Extensive lipid traffic between prokaryotic and eukaryotic pathways for lipid synthesis has been documented, although this has mostly focused on the chloroplastic import of eukaryotic lipids for MGDG, DGDG, and PG synthesis (Hurlock et al., 2018). Currently, two lipid traffic routes have been demonstrated: one is the Trigalactosyldiacylglycerol (TGD)-mediated pathway (Roston et al., 2012; Hurlock et al., 2014), and another is through plastid–ER and ER–PM membrane contact sites (Michaud and Jouhet, 2019). Chloroplasts often localize to the proximity of the plasma membrane; so far, it remains unclear whether chloroplast–PM contact sites exist, which could serve as a crossroad for lipid traffic. It is worth noting that the overexpression of *FAD4* (*OX-FAD4_2*) led to an increased PG (36:7) content in whole leaf lipid but not in the plasma membrane (**Figures 4B, D**), suggesting that more PG (36:7) could be retained in the chloroplast. We speculate that some unidentified factors may restrict its traffic from the plastid to the plasma membrane. In the *fad4–2* plasma membrane, PG (36:7) was significantly reduced (**Figure 4B**), and other PG molecular species, including PG34:2, PG34:3, PG34:4, PG36:1, and PG36:3, were also lower compared to the WT control, and collectively contributed to reduced total PG contents in the plasma membrane (**Figure 4A**).

### 3.6 AtANN2 and AtANN4 proteins showed higher association with *fad4* plasma membrane

Based on the HS-induced acute [Ca^2+^]_Cyt_ signature in the *fad4* mutant (**Figure 1**), we reasoned that the candidate calcium channels should meet the following criteria: 1) they must be located in the plasma membrane; 2) they must be activated rapidly (with a time frame of ~1–2 s) upon HS; 3) their overexpression should enhance HS-induced acute [Ca^2+^]_Cyt_ spike, and overexpressing plants should exhibit higher thermal tolerance; and 4) knockout mutants should reduce HS-induced acute [Ca^2+^]_Cyt_ spike, and mutant plants show reduced thermal tolerance. By now, multiple calcium-permeable channels in the plasma membrane have been identified and characterized, including glutamate receptor-like channels (GLRs), CNGCs, mechanosensitive Ca^2+^ channels (MCA1/MCA2), and annexins (Wdowiak et al., 2024; Ding et al., 2024; Kudla et al., 2010; Vincill et al., 2012; McAinsh and Pittman, 2009). GLRs are amino acid-gated Ca^2+^-permeable channels and mediate a [Ca^2+^]_Cyt_ spike with a lag period of ~5–20 s following agonist application (Vincill et al., 2012; Stephens et al., 2008). Although some members of GLRs are involved in cold tolerance (Gong et al., 2019; Michard et al., 2011), none have been reported to participate in HS response. Unlike GLRs, several members of CNGCs, including CNGC2, CNGC4, CNGC6, and CNGC16, have been demonstrated to participate in HS response (Tian et al., 2019; Chin et al., 2013; Finka et al., 2012; Gao et al., 2012; Niu et al., 2020; Tunc-Ozdemir et al., 2013). As ligand-gated channels, CNGCs are only active when cAMP or cGMP is bound to its CNBD; thus, a lag period always exists between stimuli and CNGC-mediated [Ca^2+^]_Cyt_ spike, during which a transient elevation of cAMP or cGMP occurs ahead of the appearance of [Ca^2+^]_Cyt_ spike. Depending on the types of stimuli, this lag period is approximately several minutes (Lu et al., 2016; Gao et al., 2012; Niu et al., 2020). Thus, GLRs and CNGCs are excluded from consideration. MCA1 and MCA2 modulate cold-induced acute Ca^2+^ influx (Mori et al., 2018; Nakagawa et al., 2007). Knockouts of MCA1, MCA2, or both mutants exhibited sensitivity to chilling and freezing (Nakagawa et al., 2007; Yamanaka et al., 2010; Mori et al., 2018). Thus, MCA1 and MCA2 are involved in CS-induced acute [Ca^2+^]_Cyt_ spike (**Figure 1**).

**Figure 5:**
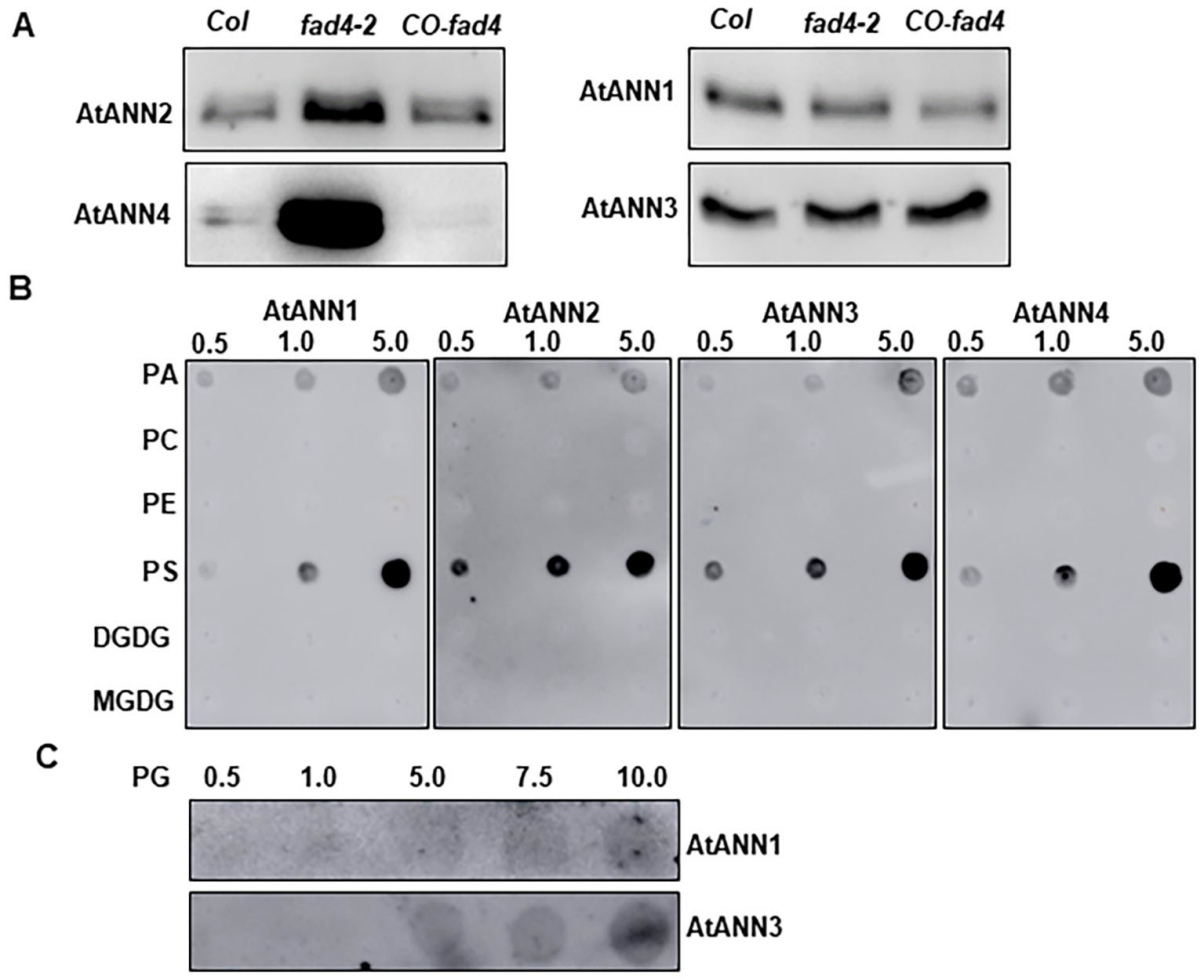
The effect of *fad4* mutation on annexin association with plasma membrane. **(A)** Annexin liposome association assay. The plasma membrane was first purified from *Col*, *fad4-2*, and *fad4–2* complementation line (*CO-fad4*), and total lipids were then isolated from purified plasma membrane and used to prepare liposomes. The amount of annexin bound to liposome was measured by Western blotting with His tag monoclonal antibody (HRP-conjugated). **(B, C)** Fat Western blotting: 0.5, 1.0, or 5.0 μg lipid standards **(B)** or 0.5–10 μg PG **(C)** was spotted onto nitrocellulose and then incubated with recombinant AtANN1, AtANN2, AtANN3, and AtANN4. PA, 1-palmitoyl-2-oleoyl-*sn*-glycero-3-phosphate; PC, 1-palmitoyl-2-oleoyl-*sn*-glycero-3-phosphocholine; PE, 1-palmitoyl-2-oleoyl-*sn*-glycero-3-phosphoethanolamine; PS, 1,2-diacyl-*sn*-glycero-3-phospho-l-serine; DGDG, digalactosyl diacylglycerol; MGDG, monogalactosyl diacylglycerol; PG, 1,2-dioleoyl-*sn*-glycero-3-phospho-rac-(1-glycerol).

Annexins are the best candidates to generate this acute [Ca^2+^]_Cyt_ spike based on the following experimental evidence: 1) annexins have been demonstrated to function as Ca^2+^-permeable cation channel (Gerke et al., 2005; Laohavisit et al., 2012); 2) both AtANN1 and AtANN4 mediate an acute [Ca^2+^]_Cyt_ spike following various stimuli including heat, radical, salt, and pathogen elicitor (Richards et al., 2014; Laohavisit et al., 2012; Ma et al., 2019; Zhao et al., 2019; Liao et al., 2017); and 3) knockouts of *atann1* or *atann2* reduce HS-induced [Ca^2+^]_Cyt_, and mutant plants are more sensitive to HS treatment, whereas the overexpression of *AtANN*s enhanced heat stress tolerance (Chu et al., 2012). These literature reports are aligned with the notion that the HS-induced acute [Ca^2+^]_Cyt_ elevation (**Figure 1**) could be mediated by annexins. To further validate this hypothesis, we first examined whether annexin abundances are affected by the *fad4* mutation. RT-qPCR demonstrated that the transcriptional levels of seven annexins (*AtANN1*–*7*) were not significantly affected by *fad4* mutation either under normal growth conditions or after HS treatment (**Supplementary Figure S4**). Since annexins are amphipathic proteins and can be cytosolic, membrane-associated, or membrane-inserted (Mortimer et al., 2008), we asked whether annexin association or insertion into plasma membranes could be affected in the *fad4* mutant. To test this possibility, we purified plasma membrane lipids from WT, *fad4-2*, and *fad4–2* complementation line (*CO-fad4*) to reconstitute liposomes. The renatured recombinant 6x His-tagged AtANN1, AtANN2, AtANN3, and AtANN4 proteins (**Supplementary Figure S2**) were used for liposome association assays. We found that AtANN2 and AtANN4 exhibited higher binding capacity toward liposomes prepared from *fad4* plasma membrane lipids, while AtANN1 and AtANN3 showed similar binding among WT, *fad4* mutant, and *CO-fad4* (**Figure 5A**). These data indicate that FAD4-mediated membrane lipid compositional changes enhanced AtANN2 and AtANN4 protein membrane association, but did not affect AtANN1 and AtANN3 membrane association, suggesting that some endogenous features of annexin isoforms could also contribute to their membrane binding capacity.

To further pinpoint the lipid species that specifically interact with annexins, various phospholipid and glycolipid standards were obtained from commercial sources, and their binding capacity toward AtANNs was analyzed via a fat Western blotting assay. We found that anionic phospholipids PS, PA, and PG, but not PC, PE, DGDG, and MGDG, specifically bound with recombinant AtANN1, AtANN2, AtANN3, and AtANN4. PS showed the highest affinity, followed by PA and then PG (**Figures 5B, C**).

## 4 Discussion

### 4.1 HS-induced acute calcium influx could be mediated by plasma membrane-ANN2/ANN4 module

**Figure 6:**
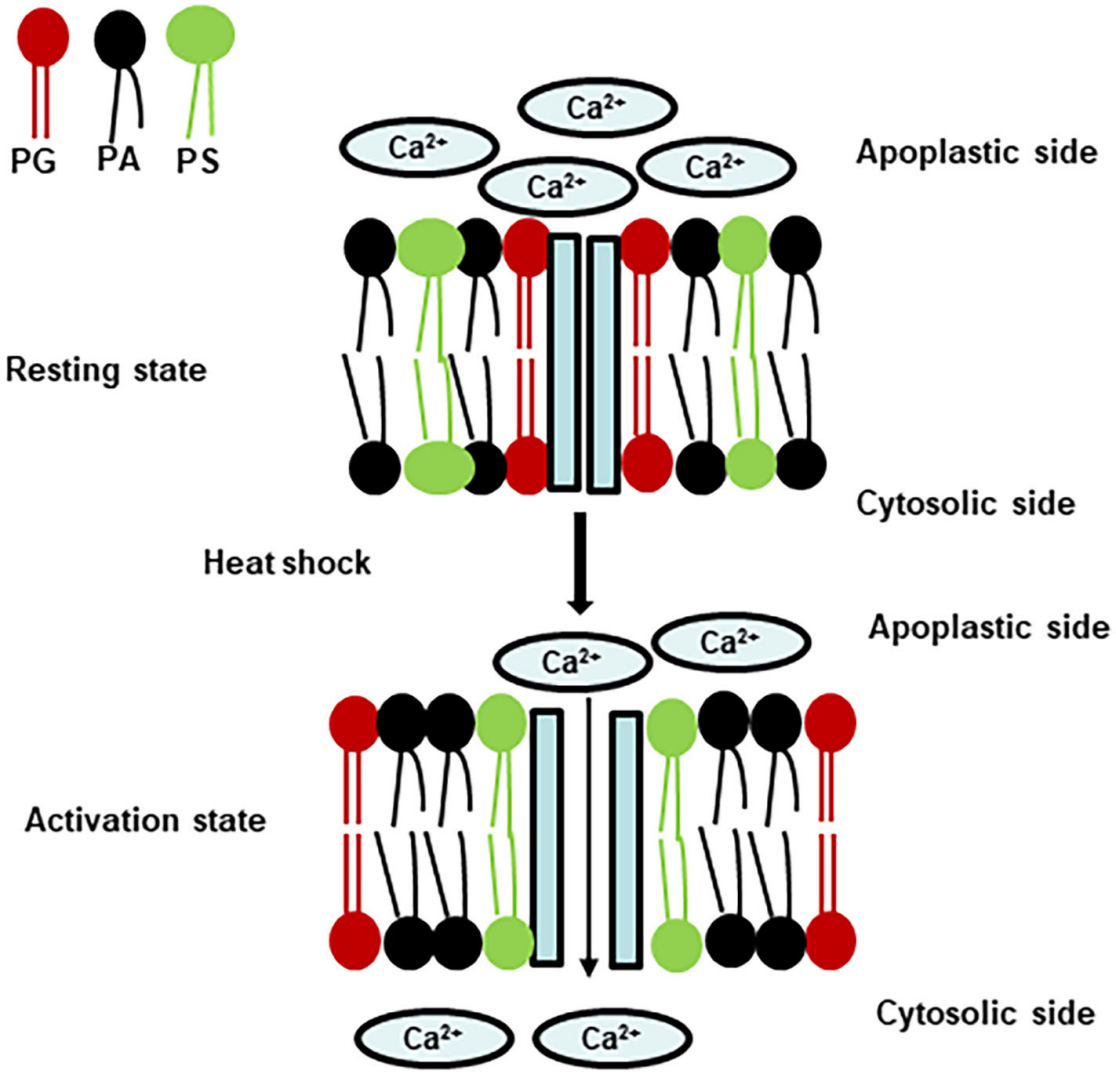
A proposed working model that heat shock activates annexin-permeable calcium channel by affecting lateral membrane movement of anionic phospholipid. PS, phosphatidylserine; PA, phosphatidic acid; PG, phosphatidylglycerol.

Plasma membrane and Ca^2+^ ions have been proposed as both heat and cold sensors, respectively (Ma et al., 2015; Ding et al., 2019, Ding et al., 2024; Saidi et al., 2011, Saidi et al., 2009; Gong et al., 1998; Liu et al., 2005; Wu and Jinn, 2010). Considering that heat increases membrane lipid kinetic energy and molecular motion, while cold shows opposite effects, it is expected that plants could use different sensors for heat and cold in order to define downstream signaling specificity. Based on previous literature reports and this work, even though both cold and heat can induce similar acute [Ca^2+^]_Cyt_ spike (**Figure 1**), the mediating calcium channels are different: cold-induced acute [Ca^2+^]_Cyt_ spike is likely mediated by MCA1, MCA2, and ANN1 (Mori et al., 2018; Liu et al., 2021; Ding et al., 2024), while the HS-induced acute [Ca^2+^]_Cyt_ spike is mediated by ANN1, ANN2, and ANN4 (Wang et al., 2015; Liao et al., 2017). Even though ANN1 is involved in CS- and HS-induced acute [Ca^2+^]_Cyt_ spike, its binding to plasma membranes was not affected in the *fad4* mutant (**Figure 5A**, right panel). In contrast, the ANN2 and ANN4 association with plasma membranes was enhanced (**Figure 5A**, left panel). These data are in accordance with our observation that the acute [Ca^2+^]_Cyt_ spike in the *fad4* mutant was elevated by HS but not CS (**Figure 1**). Although significantly more ANN2 and ANN4 were inserted into *fad4* plasma membrane compared to WT (**Figure 5A**), the resting [Ca^2+^]_Cyt_ amplitudes in WT and *fad4–2* mutants only exhibited a marginal difference (0.007 vs. 0.011 μM), and a significant [Ca^2+^]_Cyt_ increase in *fad4–2* was observed only after HS stimulation (**Figure 1**). These data suggest that *fad4*-mediated plasma membrane lipid compositional changes also facilitate ANN2 and ANN4 channel activation in response to HS but not CS. To accommodate these findings, we propose a working model in **Figure 6** to explain annexin calcium channel activation by heat shock. In this model, we hypothesized that annexins and anionic lipids form a microdomain in the plasma membrane. PG or PG (36:7) may function as a boundary lipid to the annexin oligomer, probably due to its more compact structure resulting from its *trans*-double bond. This configuration could prevent direct interactions of PA or PS with annexin oligomer, thus maintaining the annexin calcium channel at the resting state. For annexin activation, the replacement of PG by PA or PS could be required to allow PS/PA direct interactions with annexins. Heat-induced local entropy increase could disrupt initial PG–annexin interaction and facilitate PA/PS–annexin direct interaction, leading to calcium channel activation. This hypothesis reasonably explains the following experimental observations: 1) why the *fad4* mutant only enhances HS-induced [Ca^2+^]_Cyt_ elevation but not CS (**Figure 1**). HS could increase plasma membrane lipid molecular motion, thus disrupting the balance of annexin interactions with anionic phospholipids, especially enhancing direct interactions of annexins with PS or PA, leading to annexin Ca^2+^ channel opening and calcium influx, as we observed (**Figure 1**, right panel). In contrast, cold shock is expected to reduce plasma membrane lipid molecular motion and fix stochastic interactions between annexins and PGs. Thus, CS could not activate ANN2 and ANN4, and WT and *fad4–2* could show similar [Ca^2+^]_Cyt_ spike as we demonstrated in the left panel of **Figure 1**, **2**) Why lower levels of PG and PG (36:7) in the *fad4* mutant plasma membrane could result in annexin activation in response to heat shock (**Figure 1**, right panel). Even though plasma membrane PS and PA levels were similar between WT and *fad4–2* mutant (**Supplementary Figure S5**), the lower PG and PG (36:7) (**Figure 4**) with concurrent higher ANN2 and ANN4 present in the *fad4* plasma membrane (**Figure 5A**) could increase the chance for direct interactions of PA/PS with ANN2/ANN4 and lead to enhanced Ca^2+^ influx in response to HS as we observed (**Figure 1**, right panel). Overall, this proposed working model could explain how a physical signal, such as HS, could be converted into a chemical signal, such as [Ca^2+^]_Cyt_ spike, through its effect on plasma membrane lipid kinetics and lipid–annexin interaction. Consistent with the proposed role of PG (36:7) for heat tolerance, Burgos et al. (2011) reported that PG (36:5), a precursor for PG (36:7) synthesis, was immediately decreased following HS, which corroborates our finding that *FAD4* transcription was rapidly downregulated by HS (**Figure 3C**). Downregulation of PG (36:5) and PG (36:7) could prime an elevated calcium signaling in response to the following HS, which marks the first stage of adaptation to higher growth temperature.

### 4.2 The chloroplasts could contribute to HS-induced intracellular calcium release

The ruthenium red inhibition results suggest that the *fad4* mutation also enhanced intracellular calcium release in response to HS or H_2_O_2_ stimuli (**Figure 1**, right panel; **Figure 2**). However, our current data cannot pinpoint the contributing intracellular sub-compartments. Chloroplasts could be the potential contributor to HS- or H_2_O_2_-induced acute [Ca^2+^]_Cyt_ spike for the following considerations: 1) both 16:1^t^-PG and PG (36:7) are present in thylakoid membranes, and their contents were significantly reduced in the *fad4* mutant (**Figure 4**; Chen et al., 2023; Gao et al., 2009); 2) chloroplasts are important intracellular calcium storage sites and contribute to extracellular calcium-induced [Ca^2+^]_Cyt_ transients (Han et al., 2003; Tang et al., 2007; Nomura et al., 2008; Weinl et al., 2008); and 3) multiple Ca^2+^-permeable channels present on chloroplast membranes, including annexins, CASTOR, POLLUX, and Ca^2+^-sensing receptor (CAS), which are capable of releasing Ca^2+^ in response to various stimuli (Seigneurin-Berny et al., 2000; Rudella et al., 2006; Friso et al., 2004; Sai and Johnson, 2002; Imaizumi-Anraku et al, 2005; Nomura et al., 2008; Weinl et al., 2008; Lenzoni and Knight, 2019). It will be interesting to investigate whether the *fad4* mutation affects these Ca^2+^ channels’ association or activation on the chloroplast membrane.

### 4.3 The roles of H_2_O_2_ in heat shock-induced acute [Ca^2+^]_Cyt_ signature

Increasing evidence suggests a mutual interplay between calcium and ROS signaling systems (Görlach et al., 2015; Ravi et al., 2023; Marcec et al., 2019). Ca^2+^ signaling-dependent ROS production has been demonstrated in a number of studies (Ma et al., 2009; Boudsocq et al., 2010), while ROS-induced Ca^2+^ signaling is documented in plant adaptation to stress (Rentel and Knight, 2004; Lecourieux et al., 2002). Since heat induces oxidative stress (Gong et al., 1997a, Gong et al., 1997b; Larkindale and Knight, 2002), and oxidative stress triggers Ca^2+^ spikes (Price et al., 1994), one could reason that HS-induced [Ca^2+^]_Cyt_ spike could be mediated by H_2_O_2_. In this study, the HS- and H_2_O_2_-induced [Ca^2+^]_Cyt_ spikes were compared (**Figure 1**, right panel; **Figure 2**), and clear differences in their calcium signatures were observed: HS induced a rapid [Ca^2+^]_Cyt_ spike lasting for 20 s and then decays quickly (**Figure 1**, right panel). In contrast, in addition to inducing a small transient [Ca^2+^]_Cyt_ spike similar to HS, H_2_O_2_ also induced a second prolonged large peak, which decays much more slowly (**Figure 2**). These observations suggest that H_2_O_2_-induced [Ca^2+^]_Cyt_ spike may involve calcium-activated calcium release, which has been reported in other stimulus-induced [Ca^2+^]_Cyt_ signatures (Ward and Schroeder, 1994; Bewell et al., 1999). Since the H_2_O_2_-induced acute peak (the first small peak) was also significantly reduced in the *fad4* mutant (**Figure 2A**), we speculate that this first small peak could be mediated by annexins, which are also activated by H_2_O_2_; the annexin activation by H_2_O_2_ has been demonstrated by Richards et al. (2014), even though the underlying activation mechanisms remain unclear. Interestingly, when GdCl_3_ treatment abolished this transient acute peak difference between WT and *fad4–2* mutant, this also abolished the difference for the second prolonged large peak (**Figure 2B**), suggesting that this acute calcium wave is necessary to activate unidentified calcium channels that mediate this second calcium wave. Recently, Wu et al. (2000) demonstrated that H_2_O_2_ is sensed by an LRR receptor kinase HPCA1, resulting in its autophosphorylation, which activates unidentified Ca^2+^ channels leading to [Ca^2+^]_Cyt_ spike. Marcec and Tanaka (2022) also reported that cytosolic calcium elevation is required to initiate and regulate apoplastic ROS production generated by respiratory burst oxidase homologs (RBOHs). Based on these findings, we speculate that exogenous H_2_O_2_ application may first activate annexin, leading to the acute [Ca^2+^]_Cyt_ spike. This first calcium wave may lead to an endogenous H_2_O_2_ burst, which is sensed by the HPCA1 receptor, leading to the activation of unidentified calcium channels to generate the second prolonged large peak. Such a scenario raises another question: if annexins mediate both HS- and H_2_O_2_-induced acute calcium spike, why can H_2_O_2_ induce a second calcium wave but HS cannot (**Figures 1**, 2)? We tentatively speculate that two potential explanations may account for this phenomenon: 1) HS-induced acute [Ca^2+^]_Cyt_ may not lead to immediate endogenous ROS burst; 2) the activation of the unidentified Ca^2+^ channels responsible for the second calcium wave generation may require H_2_O_2_ pre-priming. Obviously, this question requires further experimental validation.

## Data Availability

The original contributions presented in the study are included in the article/**Supplementary Material**. Further inquiries can be directed to the corresponding author.

## Supplementary material

Supplementary Figure 1 RT-PCR confirmation of transcriptional complementation of *fad4* mutant by expressing wild-type *FAD4* gene **(A)** and overexpression of *FAD4* in *Col* wild-type **(B)**.

Supplementary Figure 2 Isolation of recombinant 6x His fusion of AtANN1, AtANN2, AtANN3, and AtANN4. Recombinant proteins were expressed in *E. coli* ROSETTA (DE3), and purified under denaturing conditions.

Supplementary Figure 3 The expression of heat shock factors among WT (*Col*), *fad4-2*, and *fad4–3* mutant plants. *Col*, *fad4-2*, and *fad4–3* seeds were germinated on 0.5x MS medium, one-week-old seedlings were moved to 37 °C chamber for 1 h for heat shock (HS) treatment, then recovered at 22 °C for 2 h before tissues harvesting, RT-qPCR was applied to quantify gene expression. Data was expressed as mean ± SD (n = 3).

Supplementary Figure 4 *fad4* mutation did not significantly affect the transcription levels of annexin family genes. *Col*, *fad4-2*, and *fad4–3* seeds were germinated on 0.5x MS medium under continuous light for 7 days, seedlings were moved to 37 °C growth chamber for 1 h, then recovered in 22 °C growth chamber for 2 h before harvesting the tissues for RNA isolation. The control plants were kept in 22 °C growth chamber. RT-qPCR was performed to quantify gene expression. Data was expressed as mean ± SD (n = 3). *AtANN5* expression was not detected in this study.

Supplementary Figure 5 *fad4* mutation did not alter PS and PA contents in plasma membrane. Plasma membrane was purified from *Col*, *fad4-2*, and *OX-FAD4* plants by two phase separation, total plasma membrane lipids were isolated from purified plasma membrane, lipids were analyzed by electrospray ionization tandem mass spectrometer using sequential precursor and neutral loss scans. Four biological replicates were applied; data were expressed as mean ± standard deviation (n = 4).

Supplementary Table 1 Primer sequences used for RT-qPCR in this study.

Supplementary Table 2 Primer sequences of *AtANN1*, *AtANN2*, *AtANN3*, and *AtANN4*.
