# Supplementary material for: Arabidopsis fad4 mutant analysis provides insights into thermo sensing within plant plasma membrane
Source: Front Plant Sci. 2026 Jan 19;16:1688284. doi: 10.3389/fpls.2025.1688284 (PMC12861913; doi:10.3389/fpls.2025.1688284)
Supplement: Supplementary Figure 1 — RT-PCR confirmation of transcriptional complementation of fad4 mutant by expressing wild-type FAD4 gene (A) and overexpression of FAD4 in Col wild-type (B). [file Supplementaryfile1.pdf]

frontiers

Supplementary Material

1 Supplementary Figures and Tables

1.1 Supplementary Figures

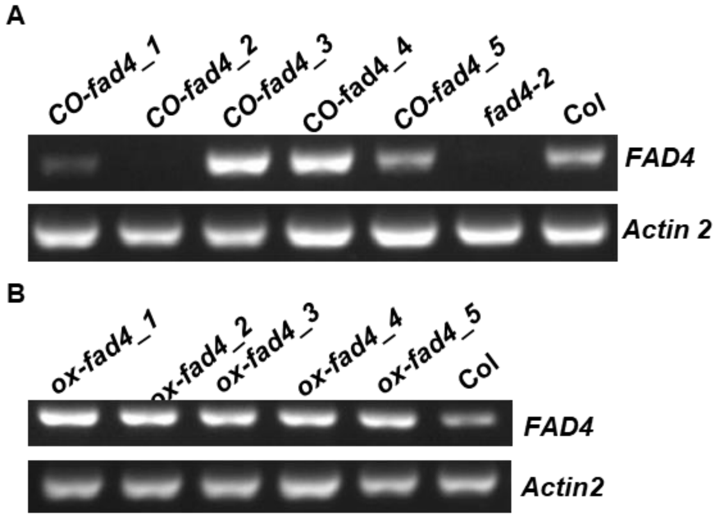

Figure S1. RT-PCR confirmation of transcriptional complementation of *fad4* mutant by expressing wild-type *FAD4* gene (A) and overexpression of *FAD4* in *Col* wild type (B).

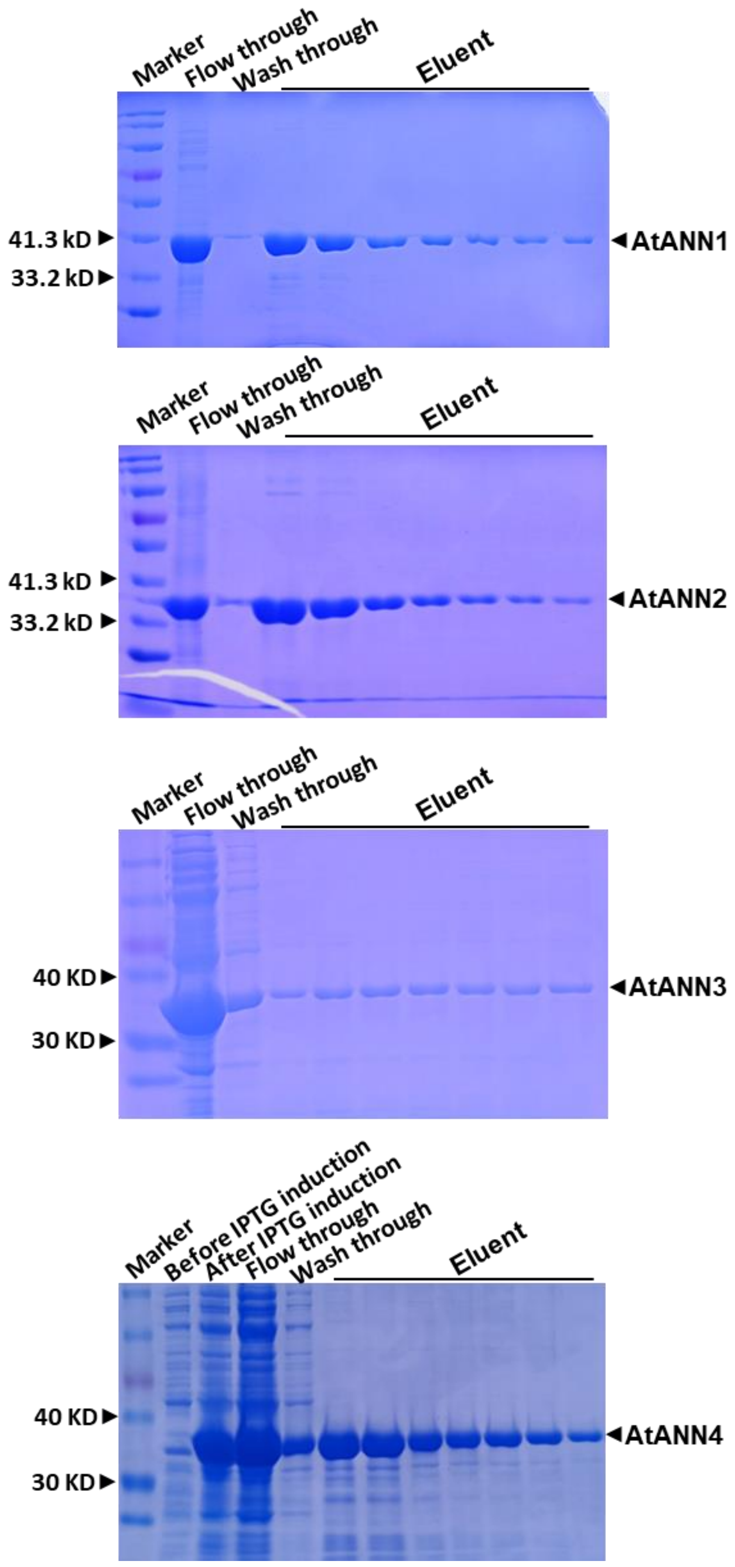

Figure S2. Isolation of recombinant 6x His fusion of AtANN1, AtANN2, AtANN3, and AtANN4. Recombinant proteins were expressed in E.coli ROSETTA (DE3), and purified under denature condition.

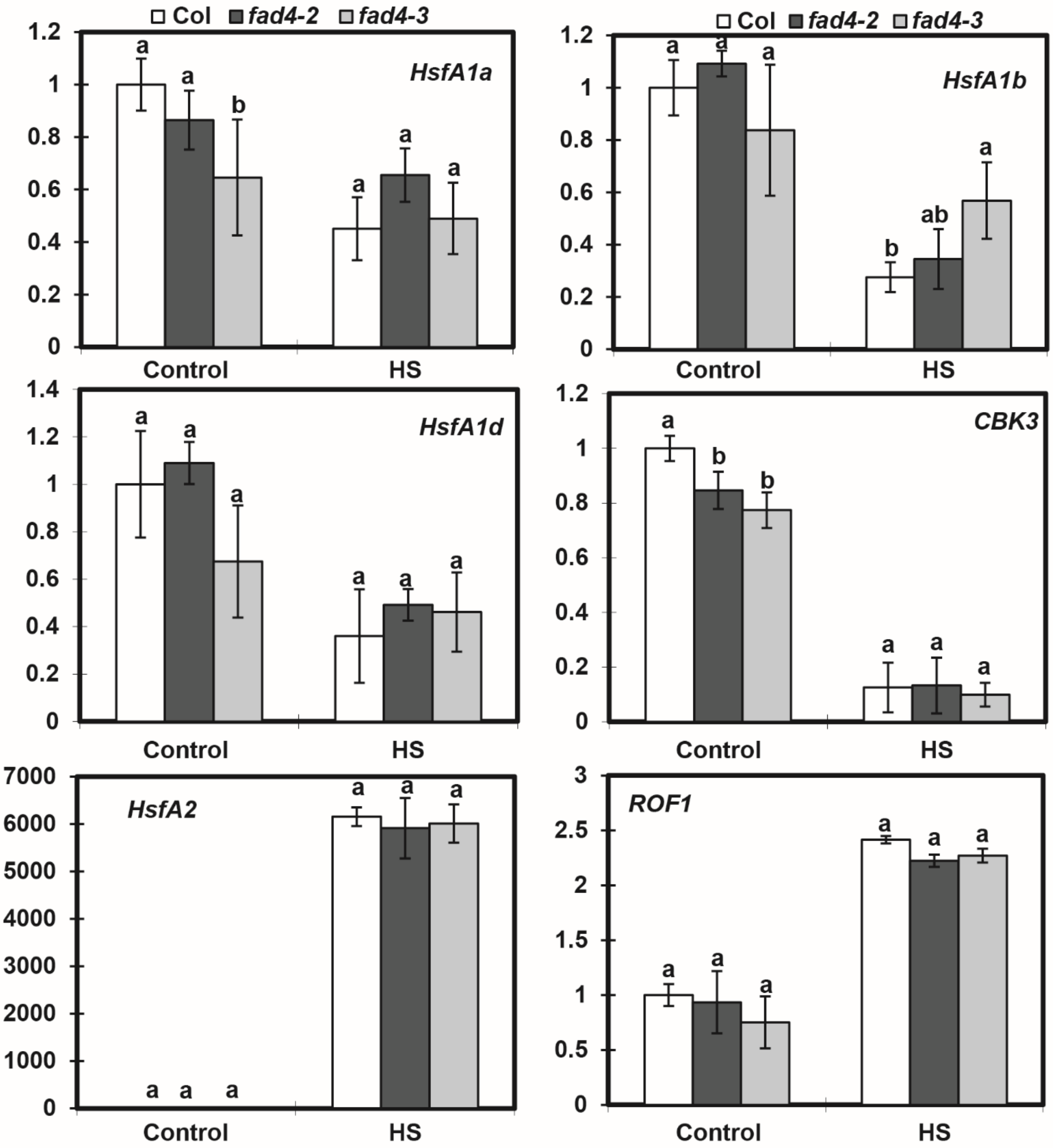

Col
fad4-2
fad4-3
HsfA1a
HsfA1b
HsfA1d
CBK3
HsfA2
ROF1
Control
HS
a
b
ab
1.2
1
0.8
0.6
0.4
0.2
0
1.4
7000
6000
5000
4000
3000
2000
1000
3
2.5
2
1.5
0.5

Figure S3. The expression of some heat shock factors was not affected in *fad4* mutant plants. For heat shock treatment, seven-day-old plants grown on ½ MS medium were treated at 22 °C (CK) or 37 °C (HS) for 1 h, then recovered at 22 °C for 2 h before tissues harvesting for total RNA isolation. RT-qPCR was applied to monitor gene expression. HS, heat stress.

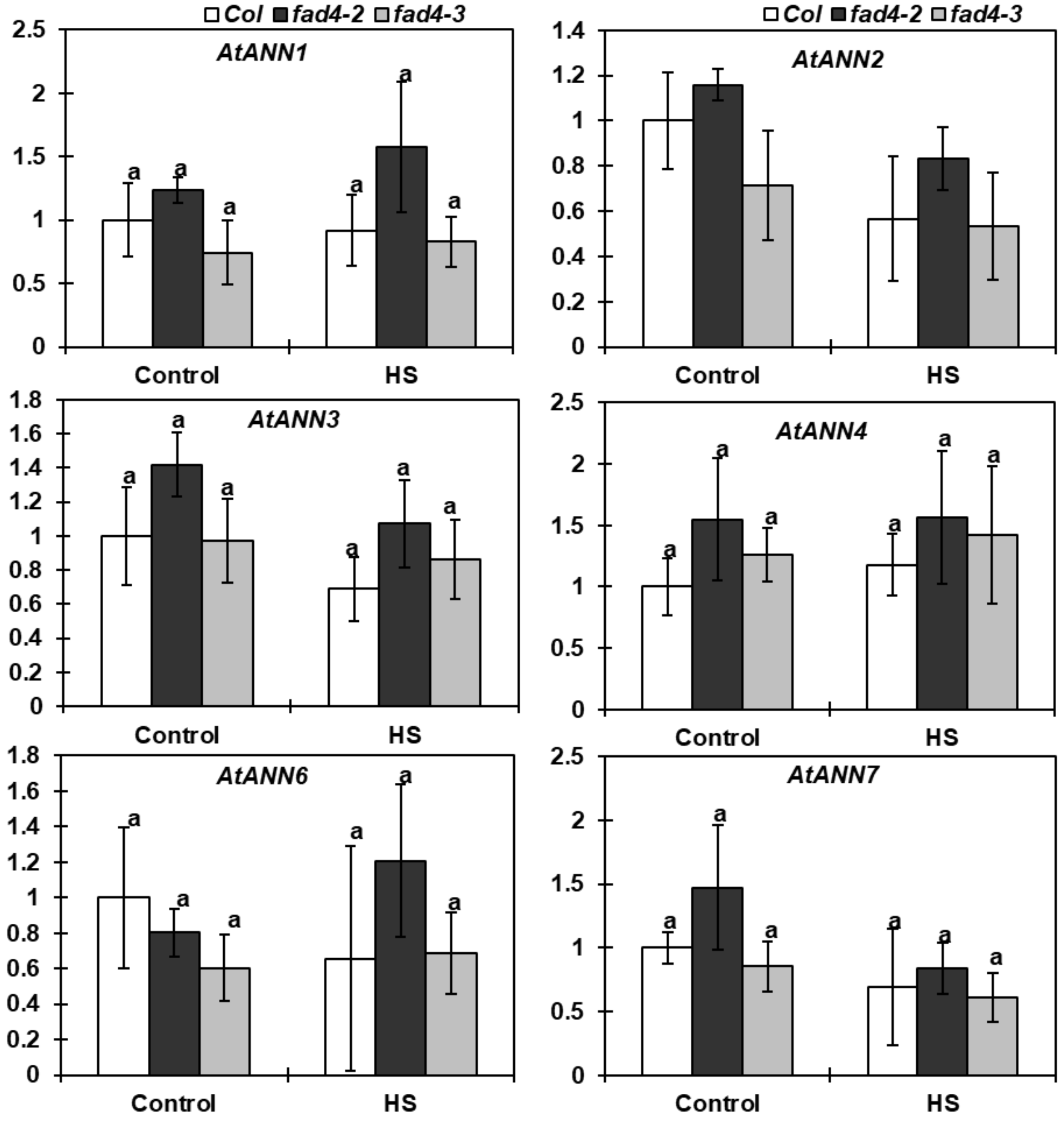

Figure S4. *fad4* mutation did not significantly affect the expression levels of annexin family genes. *Col*, *fad4-2* and *fad4-3* seeds were germinated on 0.5x MS medium under continuous light for 7 days, seedlings were moved to 37 ℃ growth chamber for 1 h, then recovered in 22 ℃ growth chamber for 2 h before tissue harvesting for RNA isolation. The control plants were kept in 22 ℃ growth chamber. RT-qPCR was performed to monitor gene expression, three biological replicates were used. *AtANN5* expression was not detected. Data was expressed as mean ± SD (n = 3). HS, heat stress.

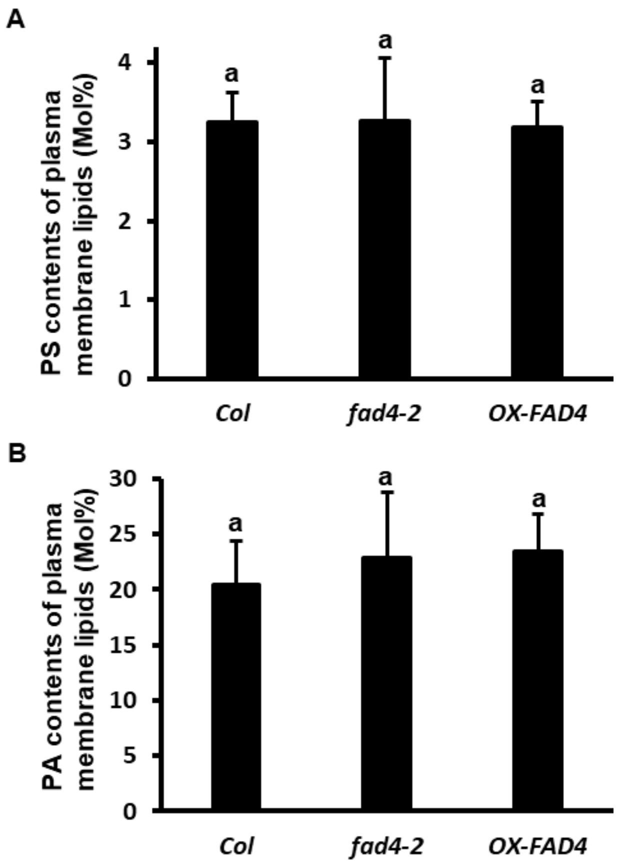

Figure S5. *fad4* mutation did not affect PS and PA levels in plasma membrane. Plasma membrane was purified from *Col*, *fad4-2*, and *OX-FAD4* plants by two phase separation, total plasma membrane lipids were isolated from purified plasma membrane, lipids were analyzed by electrospray ionization tandem mass spectrometer using sequential precursor and neutral loss scans. Four biological replicates were applied, data were expressed as mean ± standard deviation (n = 4).

1.2 Supplementary Tables

Table S1. Primer sequences used for RT-qPCR in this study

**Primer Name**	**Primer Sequence 5′→3′**
HSFA1a_F	CTCCAGCTTTGTTCGCCAG
HSFA1a_R	TCTCCGGCTTATCTTCTTCAATAG
HSFA1b_F	ACTTCTCCAGCTTCGTCAGACAG
HSFA1b_R	CTTGAGTTTGTTGCTGATTCTGCT
HSFA1d_F	GACAGCTTAATACCTATGGTTTCAGG
HSFA1d_R	CTGATGTCCCTGTCCCTGTC

Primer Name	Primer Sequence 5′→3′
CBK3_F	CTTGCATCGGAACTCGGACTAG
CBK3_R	CTAAGCTTTTTGTAAGGTCCGAGAAG
HSFA2_F	CGTCAGCTCAATACTTATGGATTCAG
HSFA2_R	CACATGACATCCCAGATCCTTG
ROF1_F	TCCAAGAGATATGAGAAGGCTGTCA
ROF1_R	CTTTGTGCATAGTTTTTCGGCCT
HSFA3_F	AGACAGCTTAACACTTATGGATTTCG
HSFA3_R	TGGTTGGATTGTGGTGATCG
AtANN1_F	CGAGAGAGCTATCTTGTTGTGGAC
AtANN1_R	GCAGCTGCGTTGATGTCCT
AtANN2_F	CTGGTGACCTTCGTAAGCTCTTG
AtANN2_R	CTGTGCTTTGCTTCTTGTTGTCA
AtANN3_F	TTGCAAAGGTGGTAAGAGATTCG
AtANN3_R	GGTGATAATGAAGTCCTTGTAGTCTCC
AtANN4_F	CTCTGATCTTCTTGGGGGTGTATC
AtANN4_R	GAGCCAAAGTCTCACCATAAAGG
AtANN5_F	GACCGAAGCAGGACTCATTTG
AtANN5_R	CGCCGTGTCATCTGTTCCTA
AtANN6_F	ATCCGAAAGCTATTGGTTCCTCT
AtANN6_R	GTAAGCCTTCTCAGTGATCTTCTTGTG
AtANN7_F	AGACATTCGAAAGCTCTTGGTACC
AtANN7_R	GTAGTGATTGAGAGTTGCGCTGAT
AtUBQ_F	GATGGTCGTACTTTGGCGGATTAC
AtUBQ_R	AGACGCAACACCAAGTGAAGGG

Table S2. Primer sequence for *AtANN1*, *AtANN2*, *AtANN3* and *AtANN4*.

Primer name	Primer sequence
ANNAT1-EcoRI F:	5’-CCGAATTCATGGCGACTCTTAAGGTTTCTGATTCTG-3’
ANNAT1-XhoI R:	5’-CCCTCGAGTTAAGCATCATCTTCACCGAGAAGTG-3’
ANNAT2-EcoRI F:	5’-CCGAATTCATGGCGTCTCTCAAAGTCCCAAGCA-3’
ANNAT2-XhoI R:	5’-AACTCGAGTCAAGCATCGCCATGTCCGAGAAGAG-3’

ANNAT3-EcoRI F:	5’-CCGAATTCATGGCCACCATTAGAGTACCAAACGA-3’
ANNAT3-XhoI R:	5’-GGCTCGAGTCAGATTTTGGATCCAAGTAAGGTGA-3’
ANNAT4-EcoRI F:	5’-CCGAATTCATGGCTCTTCCTCTCGAGCTCGA-3’
ANNAT4-SalI R:	5’-CGGTCGACTCAATCGGATTTGGAGAGAAGTGT-3’
